# Human-AI co-design for clinical prediction models

**DOI:** 10.1038/s41746-026-02838-5

**Published:** 2026-06-06

**Authors:** Jean Feng, Avni Kothari, Patrick Vossler, Andrew Bishara, Lucas Zier, Newton Addo, Aaron Kornblith, Yan Shuo Tan, Chandan Singh

**Affiliations:** 1 University of California, San Francisco, CA USA; 2 National University of Singapore, Singapore, Singapore; 3 Microsoft Research, Redmond, WA USA

**Keywords:** Computational biology and bioinformatics, Health care, Mathematics and computing, Medical research

## Abstract

Developing safe, effective, and practically useful clinical prediction models (CPMs) traditionally requires extensive collaboration between clinical experts, data scientists, and informaticists to refine the many small but critical details of the model building process. When incorporating unstructured clinical notes, this challenge magnifies, as notes essentially contain an infinite number of concepts that can be used for modeling. We introduce HACHI, an iterative human-in-the-loop framework that uses AI agents to accelerate the development of fully interpretable CPMs from clinical notes, where CPMs are defined as linear models of yes-no questions. HACHI alternates between an AI agent that uses statistical tools and embedded knowledge to explore candidate concepts and domain experts who provide feedback to the AI agent. The framework optimizes for transparency, steerability, and reciprocal learning to ensure effective collaboration between the clinical AI team and the AI agent. In two real-world prediction tasks (acute kidney injury and traumatic brain injury), HACHI outperforms existing approaches, discovers clinically relevant concepts, and improves model generalizability across clinical sites and time periods. HACHI also highlights the critical role of human oversight, such as in directing the AI agent to explore new concept categories, adjusting concept granularity, and identifying data bias and leakage.

## Introduction

Clinical prediction models (CPMs) translate routinely collected clinical information into structured predictions, enabling clinicians to apply shared expertise in a more consistent manner across patients. While there is growing interest in highly complex black-box CPMs, simple fully-interpretable CPMs, such as rule-based scores like SOFA for ICU mortality^1^ or PECARN and NEXUS for traumatic brain injury^2^, remain the most popular in clinical practice due to a number of major advantages. First and foremost, these models can be easily implemented by clinicians at the bedside using simple mental arithmetic or a quick chart evaluation, which facilitates their integration into the clinical workflow^3,4^. Furthermore, fully-interpretable models can be thoroughly audited by domain experts and, when necessary, modified to improve reliability, which promotes user trust^5,6^. Consequently, when simple, fully-interpretable CPMs both meet clinical standards and achieve high performance, these tools are widely used by the clinical community^7–10^.

Despite their utility, very few such CPMs have achieved the level of performance and clinical sophistication needed for widespread clinical adoption^11–13^. The main challenge is that “the devil is in the details”—a CPM is determined by the *numerous* details and decisions made in the CPM learning process, including which learning procedure was used, how the data was generated and compiled, which features are included, and how the model was evaluated^14–16^. All of these factors must be carefully chosen, which generally requires a lengthy and laborious collaboration between clinicians and data scientists to iterate on the model learning procedure so that the final CPM both satisfies complex clinical requirements and meets desired levels of performance^1,17^. To make matters worse, maximizing model performance often requires extending beyond tabular data in the Electronic Health Record (EHR) and analyzing unstructured clinical notes, as the latter often contains the most relevant and predictive features^18–20^. However, clinical notes cover an essentially *infinite* number of concepts; for example, the concept of *smoking* can be characterized by whether a patient currently smokes, has a history of smoking, is trying to quit smoking, and many more. As such, it is infeasible for clinical AI teams to manually explore all the possible concepts for inclusion in the CPM learning process.

Recent developments in artificial intelligence (AI) suggest that AI agents—here defined as encapsulated LLMs that integrate reasoning with tool-calling capabilities, including tools for model fitting and performance evaluation—can efficiently navigate and optimize many of these critical design decisions for learning fully-interpretable CPMs. Indeed, recent research has shown that because large language models (LLMs) can now mimic complex clinical reasoning and accurately extract concepts from clinical notes^21–23^, AI agents can now analyze clinical notes, brainstorm useful features to include in a CPM, convert these features into simple tabular elements, and apply statistical tools to fit models^24–28^. To find concepts in clinical notes that are most predictive of the target of interest, these methods have an AI agent iteratively explore the infinite space of concepts based on its clinical knowledge, which can reveal novel predictive signals that are difficult to identify upfront^28,29^. However, existing works only study the AI agent working in isolation. Without external guidance from domain experts, the AI agent may learn concepts that are overly simplistic or lack clinical credibility, leading to CPMs that are promising but lack the clinical sophistication necessary for translation into clinical practice.

This work studies how clinical AI teams can collaborate with AI agents to improve the CPM learning process. This cannot be achieved simply through one-time prompt tuning because of the so-called “gulf of envisionment”^30,31^. In the initial stage of building a CPM, the clinical AI teams often lack alignment and clarity on the exact task specifications, so it is often unclear how to best instruct an AI agent to achieve the team’s goals. To close this “gulf of envisionment,” the team must analyze results and reasoning traces from the AI agent and then clarify to the agent on what specific data to analyze, how exactly the outcome should be defined, what concepts to explore, what statistical tools to use, and more. This requires an iterative, collaborative process, in which the human experts gradually understand the capabilities of the AI agent, gain clarity in how the task should be specified, and learn how to align the AI agent to learn the best possible CPM. Through this process, team members can also build trust in the AI agent’s learning process and, ultimately, the resulting CPM.

**Fig. 1:**
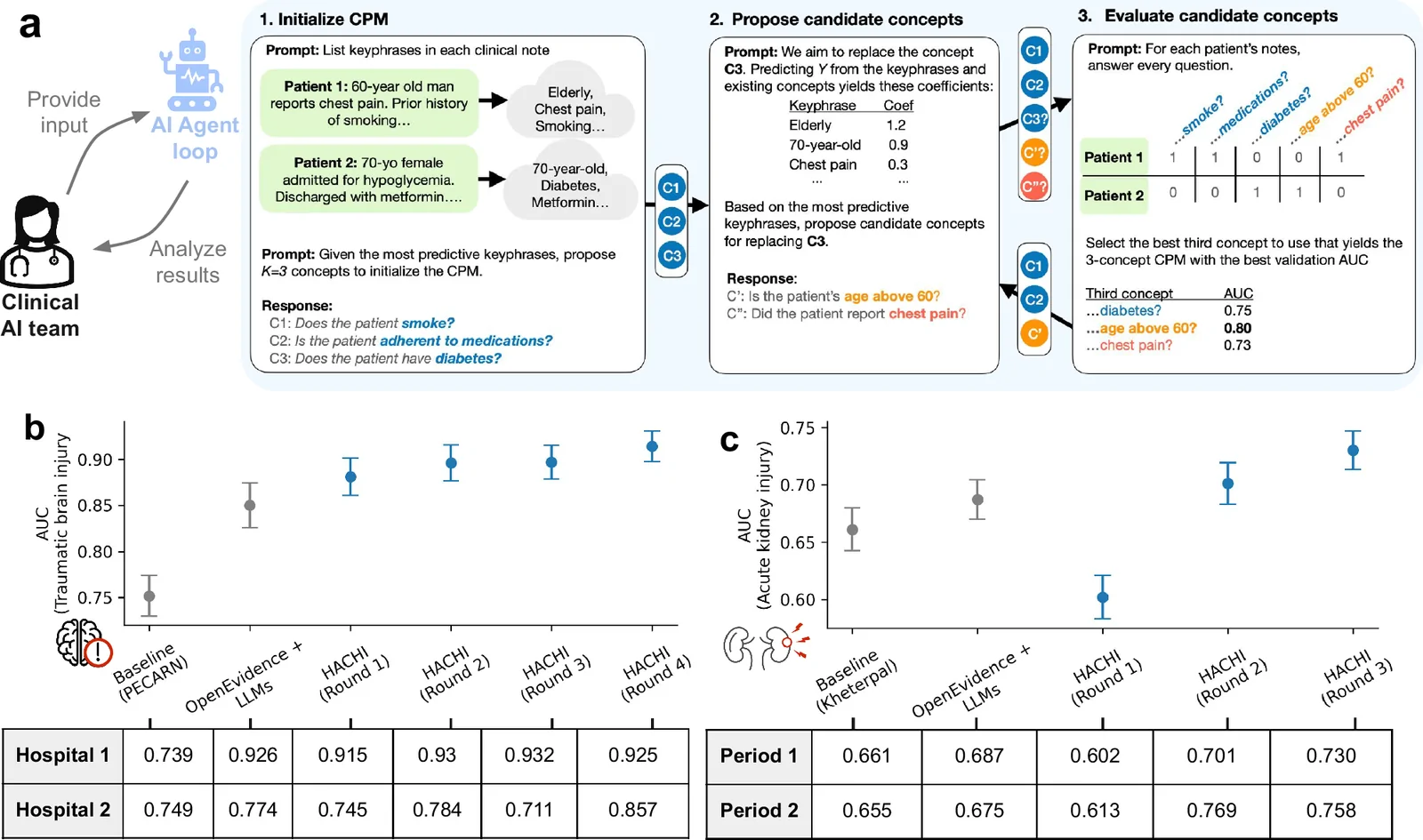
The HACHI framework uses LLMs with humans-in-the-loop to build an effective clinical prediction model (CPM). **a** HACHI is composed of an outer loop, where the clinical AI team provides guidance and feedback to an AI agent on how to learn a CPM, and an inner loop, where the AI agent follows instructions from the clinical AI team and uses its reasoning and tool-calling abilities to find *k* concepts that maximize the CPM's predictive accuracy. Concepts in a CPM (shown in the colored circles with the prefix C) are formally defined as yes/no questions. The inner AI-guided CPM learning procedure is broken down into three steps: 1. Initialize the CPM by brainstorming clinical concepts from keyphrases extracted out of clinical notes; 2. Propose candidate concepts by analyzing which keyphrases are most associated with the outcome of interest; 3. Evaluate the candidate concepts by annotating each concept and selecting the best-performing concept(s). Steps 2 and 3 are repeated until convergence. Each round, the clinical AI team analyzes the results from the AI-guided CPM learning procedure and provides feedback on how the procedure can be improved, such as by modifying the prompts and clarifying which concepts are and are not of interest. HACHI improves over baselines and across rounds for two real-world clinical prediction tasks: **b** diagnosis of traumatic brain injury (TBI), where performance is evaluated in terms of AUC with respect to the overall population (shown in plot) and stratified across two sites (shown in table). **c** Development of acute kidney injury (AKI), where performance is evaluated in an internal validation set (Period 1, shown in plot and table) and a later, temporally disjoint test dataset (Period 2, shown in table). Error bars show standard errors.

To this end, we introduce a H uman+A gent C o-design framework for H ealthcare I nstruments (HACHI). We name this framework HACHI in reference to Hachikō (1923–1935), an Akita dog renowned for his loyalty, to highlight how clinical teams must iteratively train and align their AI agent, much like how dog owners must iteratively train their faithful companions. In HACHI, the role of the AI agent is to conduct the numerous and often tedious lower-level reasoning tasks (e.g., extracting information from clinical notes, brainstorming candidate concepts, and fitting statistical models) to learn the best-performing CPM per instructions from the clinical AI team. On the other hand, the role of the clinical AI team is to analyze results from each round using their higher-level design and clinical reasoning to provide iterative targeted feedback on how the AI agent could improve its process (Fig. 1). The key contributions of HACHI is that it (1) provides a formal structure necessary for human-AI co-design of CPMs and (2) optimizes three axes that are critical to the effectiveness of this collaborative process: *transparency* of the CPM and its entire learning procedure, *steerability* of the AI agent by the clinical AI team, and ultimately *reciprocal learning*^32,33^—where the clinical AI team and AI agent learn from each other. We apply HACHI to two real-world clinical risk-prediction tasks—acute kidney injury and traumatic brain injury—to evaluate the efficacy of this human+AI agent co-design process. Code is available at http://github.com/jjfenglab/HACHI.

## Results

### HACHI overview

The goal of HACHI is to learn a CPM with *k* concepts that are interpretable, clinically reasonable, and maximize predictive performance, given a dataset of clinical notes and corresponding labels. Concepts are defined as answers to yes/no questions, such as “Does a patient have...?”. Because clinical AI teams often do not have a precise definition of this high-level goal at the beginning of a project, HACHI involves multiple rounds of fitting CPMs, each time with an “inner” AI-powered CPM learning procedure that is precisely specified but potentially imperfect. The clinical AI team is the “outer” loop that gathers new insights from each round, which are then used to improve how the inner AI-powered CPM learning procedure is run. After multiple rounds, the learning procedure becomes better-aligned with the clinical AI team’s goals, resulting in a CPM with the desired characteristics. HACHI is uniquely designed to optimize for transparency, steerability, and reciprocal learning (see additional details in the Methods section).

The inner AI-guided CPM learning procedure used in this work (Fig. 1 blue box) is a greedy hill-climbing procedure based on^28^ that iteratively refines a *k*-concept CPM by cyclically traversing each concept position and replacing the incumbent concept when a better-performing concept is found. The AI agent iteratively brainstorms, evaluates, and selects CPM concepts per the following steps:

1. *Initialize*: The AI agent creates a set of keyphrases to represent each observation, per instructions given in KeyphrasePrompt. The CPM is initialized with *k* concepts per an InitializationPrompt, and the data is partitioned into a training set and a validation set.
2. *Propose candidate concepts:* Using the training data, the AI agent fits a statistical model to identify the top keyphrases associated with the outcome. Combining this list with its prior knowledge, the AI agent proposes candidate concepts in the form of yes/no questions for inclusion in the CPM, per instructions given in ProposalPrompt.
3. *Evaluate candidate concepts*: For each observation, the AI agent reviews the clinical notes and extracts the value of each candidate concept per instructions given in ExtractionPrompt. For each candidate concept, the AI agent fits a CPM on the training data and evaluates its performance on the validation data. The candidate concept with the best-performing CPM is selected.
4. *Iterate*: Repeat steps 2–4 until convergence or until a maximum number of iterations is reached.

**Fig. 2:**
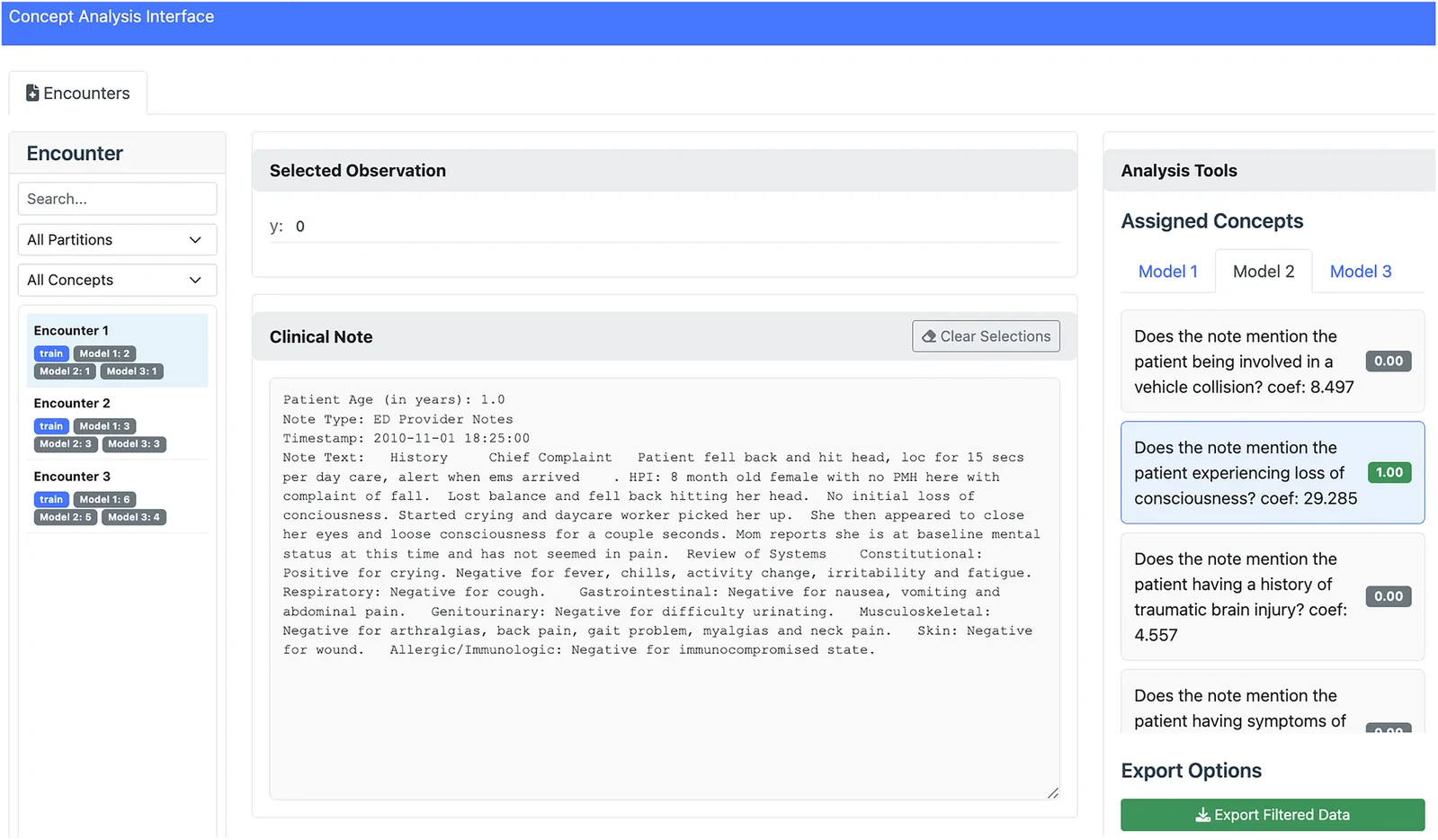
PHI-compliant web interface for auditing the AI-agent CPM learning procedure. It shows (i) the learned factors, (ii) the LLM concept annotations at a patient/note level, (iii) which patients received an incorrect prediction, and (iv) the performance of the CPM on a held-out test set.

The outer loop involves the clinical AI team providing feedback to the AI agent, by refining the hyperparameters of this procedure. The team’s key lever is through modification of free-text prompts given as instructions to the AI agent, which has the advantage of being readable by any human, even those with limited AI expertise; a default set of prompts is provided to initialize the process. Nevertheless, the team has access to other levers as well, such as modifying which observations are included in the data, developing and/or adding new statistical tools for the AI agent to use, and deciding how samples are weighted. To help teams come up with feedback for each round, a Protected Health Information (PHI)-compliant web interface is provided so that all team members can review the AI agent’s results (Fig. 2). Through this review process, the clinical AI team can decide how many rounds of HACHI to run; the following case studies found 3–4 rounds to be sufficient. Finally, based on findings from the case studies, we include a checklist (Table S6) in the Supplementary Information that clinical AI teams can reference when reviewing the outputs from the AI agent. This will help teams more systematically surface potential issues that fall into the major categories of data, concepts/variables, outcome, and training/evaluation.

Below, we describe how clinical AI teams leveraged HACHI to learn CPMs for two prediction tasks at UCSF: whether a child presenting to the Emergency Department after head trauma will be diagnosed with traumatic brain injury (TBI) and whether an adult preparing for general surgery will develop acute kidney injury (AKI) post-surgery.

### Case Study 1: Traumatic Brain Injury (TBI)

TBI is a leading cause of morbidity and mortality in children presenting to the emergency department (ED) following head trauma^34,35^. As such, it is critical to identify which children require computed tomography (CT) imaging to avoid missing TBI cases; on the other hand, ionizing radiation from CT scans can cause lethal malignancies^36,37^. To guide CT imaging decisions in pediatric head trauma, the Pediatric Emergency Care Applied Research Network (PECARN) rule is currently the most widely used CPM for identifying patients at high risk of TBI^2^. PECARN incorporates clinical signs and symptoms such as altered mental status, loss of consciousness, severe mechanism of injury, and physical examination findings (e.g., scalp hematoma, skull fractures). While the PECARN rule is designed to achieve very high sensitivity to minimize missed TBIs, its specificity remains relatively low, potentially exposing patients to unnecessary harmful radiation^38,39^.

To determine whether a better-performing CPM for TBI could be learned, a clinical AI team was assembled, including one pediatric ED clinician (A. Kornblith), an emergency clinical data analyst (N.A.), and three data scientists (J.F., C.S., A. Kothari). Given that PECARN includes only a small number of concepts, the team decided to learn a 5-concept CPM. The team collated a retrospective case-control dataset with 400 cases and 400 controls from all encounters with documented ICD codes for head trauma or traumatic brain injury (TBI) diagnoses at two academic pediatric EDs within the Benioff Children’s Hospital (Oakland and Mission Bay Campuses) between March 1, 2014, and December 31, 2024. ED Triage Notes, ED Provider Notes, and Nursing Notes were included in the analysis. To minimize the chance of data leakage, only the History & Physical section of ED provider notes were included and for the other note types, only those with timestamps prior to the encounter’s CT scan were included if a CT scan was performed. Four rounds of HACHI were completed. We describe learnings from each of these rounds below, and summarize the learned concepts, feedback, and modifications from each round in Table 1. The full set of prompts used in each round are provided in the Supplementary Information.

**Table 1:** Description of HACHI rounds for learning a 5-concept TBI prediction model

Round	Key feedback	Changes made	Learned concepts
**1**	Initial exploration with default prompts	None (baseline)	LOC (2.70), Brain bleed (1.74), Neurological event (1.58), Note mentions GCS (1.23), Seizure-free (1.10)
**2**	(1) Some concepts reflect note-writing style instead of patient characteristics; (2) Brain bleed concept suggest data leakage, investigation revealed patients with existing CT results from prior facilities	(1) Require concept questions to have prefix “Does the note mention the patient having...”; (2) Removed cases with existing TBI diagnosis or mentioned CT results	LOC (3.46), Normal GCS & age ≥2 year (1.50), Convulsions (1.04), Altered mental status (0.82), Intact cranial nerves (−0.65)
**3**	Some coefficients contradict clinical intuition	Modified greedy concept selection to require sign of estimated coefficient to match LLM’s clinical prior	LOC (4.34), History of mild TBI (1.41), Occipital hematoma (1.21), Vision changes (1.07), Memory intact (−0.53)
**4**	Model shows poor generalizability across Oakland and Mission Bay campuses due to imbalanced representation	Introduced sample weights to weight the two campuses equally	LOC (1.58), Altered mental status (0.73), Headache (0.25), Head trauma (0.08), Normal gait (−0.22)

Purple indicates features that raised concerns.

*LOC* Loss of Conciousness, *GCS* Glasgow Coma Scale.

To begin, Round 1 was run with the default template of prompts. To the surprise of the clinical AI team, this CPM already achieved a high AUC of 0.92. While this performance could be partly explained by the CPM’s overlap in concepts with PECARN (e.g., whether the patient had experienced loss of consciousness (LOC) and having a neurological event), the team also noted two major issues that could be leading to inflated performance. First, the AI agent had learned the concept “whether a note mentions Glasgow Coma Scale (GCS),” which meant that the AI agent had learned to rely on spurious correlations between the note writing style and TBI diagnosis. Second, the AI agent identified that having a “brain bleed” substantially increased the risk of TBI, but brain bleeds (i.e., intracranial hemorrhages) are typically known only *after* a patient has CT scan results. Investigating this issue of data leakage, the team found two sources: (i) half of these patients were transferred from another ED with an existing TBI diagnoses and/or imaging results and (ii) other patients had imaging results mentioned in their clinical notes even after filtering for notes with timestamps prior to the imaging order, due to note-taking patterns at the hospital. So for Round 2, the AI agent was instructed to define concept questions using the prefix “Does the note mention the patient having...”, to ensure that concepts extract attributes of the patient and not note writing style. In addition, the AI agent was instructed to remove transferred patients who already had TBI diagnoses or imaging results as well as patients whose imaging results evaded the timestamp filters, resulting in 304 remaining cases. On this clean test set, the Round 1 CPM yielded a decreased but more realistic AUC of 0.88.

Round 2’s CPM achieved a slightly improved AUC of 0.90 on the clean set of patients mentioned in Round 1. While all the learned concepts were clinically relevant, the new concern raised by the clinical AI team was that some of the learned coefficients did not match clinical intuition. For instance, having a normal GCS had a positive coefficient (i.e., increased risk), contrary to clinical expectations. After discussion with the clinical AI team, the data scientists suggested modifying the greedy concept selection process in Round 3: rather than only selecting the candidate concept based on AUC, selection additionally required the coefficient’s sign to match the LLM’s clinical prior.

With the added changes, Round 3’s CPM simultaneously had clinically reasonable concepts, directionally aligned coefficients, and maintained the same AUC of 0.90. As such, the clinical AI team decided to run one last round, where the team assessed the generalizability of the model across the two Oakland and Mission Bay campuses. This revealed a surprising gap in performance: the AUCs at the Oakland and Mission Bay campuses were 0.93 and 0.71, respectively. Investigating further, the team noted that the ratio of patients from Oakland to Mission Bay in the dataset was 3:1 and that the top feature learned by the procedure, LOC, was highly discriminative for patients at the Oakland campus but not at Mission Bay. This was clinically plausible, as the Oakland campus functions as a safety-net, pediatric Level 1 trauma referral center that sees higher-acuity and more selectively referred children, whereas the Mission Bay campus serves as a quaternary care hospital that sees a broader population. So for Round 4, the team decided to update the learning procedure to allow for sample weights, so that the two campuses could be equally weighted. Round 4’s CPM not only achieved a better overall AUC of 0.91, but also better campus-specific AUCs of 0.93 and 0.86 at Oakland and Mission Bay, respectively. The concepts in this final model are LOC, altered mental status, headache, head trauma, and having a normal gait. The concept “head trauma” was initially surprising, but analyzing the LLM’s annotations revealed that in real-world scenarios, there are a small percentage of cases (5%) where there is a suspicion of unwitnessed head trauma but no reliable patient history is available (e.g., abandoned child, unclear mechanism of injury, no chief complaint on file).

HACHI was compared against two strong baseline CPMs. First, we evaluated the performance of PECARN by extracting relevant attributes from the PECARN model^40^ using the same clinical notes. This approach yielded an AUC of 0.75, substantially below that of HACHI. Second, we simulated the common CPM-learning approach where a clinical expert outputs a list of potential risk factors and protective factors for inclusion in a CPM, but does not further refine this list. To simulate this one-time brainstorming of clinical factors, we used OpenEvidence^41^, a clinical decision-support tool that performs targeted literature searches, to generate an exhaustive list of disease-specific risk factors. These factors were then extracted from patient notes via LLM-based annotation and used to fit a CPM. Despite leveraging curated external knowledge, this pipeline achieved an AUC of 0.85, which was outperformed by HACHI. Finally, HACHI at Round 1 can be seen as an additional baseline which elicits concepts through zero-shot prompting of LLMs. We find that this zero-shot baseline (AUC of 0.88) in fact outperforms the PECARN baseline (AUC of 0.75) and the OpenEvidence baseline (AUC of 0.85). Nevertheless, all baselines are substantially outperformed by HACHI at subsequent iterations (AUC of 0.91 at the final round), suggesting that one-time knowledge elicitation, even from high-quality sources, does not capture the nuances of a specific clinical dataset and patient population.

Finally, because PECARN was designed as a rule-out tool to maximize sensitivity, we additionally compare specificity values at a fixed sensitivity of approximately 0.90 (Table S2). At this operating point, HACHI’s final-round CPM achieves a sensitivity-specificity tradeoff of 0.91–0.81, compared to 0.94-0.56 for PECARN and 0.91–0.64 for the OpenEvidence baseline, demonstrating that HACHI improves the clinically relevant tradeoff of reducing unnecessary CT scans while maintaining high sensitivity.

### Case Study 2: Acute Kidney Injury (AKI)

AKI is a common but serious postoperative complication, occurring in 2–25% of patients. When it occurs, AKI increases associated mortality and costs by two- to fivefold^42–44^. Early identification of high-risk patients enables preventive measures such as optimizing hemodynamics, avoiding nephrotoxic medications, and closer monitoring. While there are multiple well-known AKI prediction models for cardiac surgeries, there are far fewer for predicting AKI risk for lower-risk general surgeries. The most well-known interpretable AKI prediction model for General Surgery is the Kheterpal model^45^, whose predictors include patient characteristics, medications, and surgery type. Nevertheless, use of this model is limited in clinical practice, since its performance is highly dependent on the patient case mix.

To determine if a better-performing CPM for AKI could be learned, a clinical AI team was assembled with an anesthesiologist (A.B.), a clinical data analyst (J.Y.), and two data scientists (J.F. and A. Kothari). A retrospective case-control dataset was selected uniformly at random from all General Surgery patients between January 2016 and March 2024, with 800 cases and 800 controls. The outcome is defined as whether AKI develops within 7 days following surgery per the Kidney Disease Improving Global Outcomes (KDIGO) criteria (stage 1 or higher)^46^. To capture preoperative patient characteristics, the dataset consists of preoperative anesthesia notes. Given that the Kheterpal model^45^ used 11 clinical features, the team decided that a 10-concept CPM would be appropriate. Three rounds of HACHI were completed, which again are described in detail below as well as in Table 2. The full set of prompts used in each round are provided in the Supplementary Information.

**Table 2:** Summary of HACHI rounds for learning a 10-concept AKI risk prediction model

Round	Key Feedback	Changes Made	Learned Concepts
**1**	Initial exploration with default prompts focused on patient characteristics	None (baseline)	Leukocytosis (1.31), Abdominal distention (0.87), Cardiac dysfunction (0.74), Tachycardia (0.71), Swelling (0.65), Systemic infection (0.57), Renal impairment (0.53), Diabetes mellitus (0.47), No kidney disease (−0.36), Low hematocrit (−1.01)
**2**	(1) Prompts only considered patient characteristics, but surgery type is often more influential for AKI risk; (2) Learned concepts are too broad and should be more specific; (3) clinician brainstormed additional factors such as infection and need for blood transfusion	(1) Updated all prompts to include both patient and surgical risk factors; (2) Updated prompts to encourage more specific concepts, such as focusing on higher-risk factors; (3) Added clinician-suggested concepts as in-context learning examples in prompts	CKD (1.28), Exploratory laparotomy (1.03), Sepsis (0.98), Fluid retention (0.89), Respiratory condition (0.47), Need blood transfusion during surgery (0.43), High-risk surgery (0.40), Heart disease (0.37), Malignancy (0.31), Minimally invasive surgery (−1.01)
**3**	(1) Concepts too vaguely defined, which may lead to inconsistent extraction between clinicians; (2) Medication concepts missing	(1) Required concept questions to include precise definitions with examples; (2) Added medications to brainstorming prompts	CKD (1.05), Major/high-risk surgery e.g., major abdominal surgery or anticipated blood loss > 500mL (0.91), Tachycardia >100 bpm or palpitations (0.88), Active systemic infection e.g., sepsis, bacteremia, or requiring therapeutic antibiotics (0.72), Heart failure (0.64), Sleep apnea (0.51), Urgent/emergent surgery (0.50), Obesity (0.39), Hypertension (0.31), Minimally invasive surgery e.g., laparoscopic or robotic-assisted procedures (−0.74)

Blue indicates concepts learned in response to feedback from the clinical AI team.

*CKD* Chronic Kidney Disease.

To initialize, Round 1 was run with the default template of prompts for learning patient characteristics associated with the prediction target. This CPM achieved an AUC of 0.60 and identified many factors known to be associated with AKI that had been previously identified in ref. ^45^, such as renal impairment and diabetes mellitus. The method also identified features not in ref. ^45^, including hematocrit levels, leukocytosis, and cardiac dysfunction. However, the clinical AI team noted that the default prompts had directed the AI agent to only consider patient characteristics, when surgery type is often even more influential to a patient’s AKI risk. So for Round 2, all the LLM prompts were updated to instruct the AI agent to consider both patient and surgical risk factors. In addition, the clinician suggested potential risk factors that could be provided to the AI agent as in-context learning examples, such as a patient’s functional capacity, surgical duration, and whether the patient was coming from the ICU.

With the inclusion of surgical risk factors, Round 2’s CPM had a substantially higher AUC of 0.70. Some of these factors are already known (e.g., chronic kidney disease, fluid retention) and others are known but not included in commonly-used CPMs (e.g., minimally invasive surgery, active infection requiring antibiotics, requiring blood transfusion during surgery). While the performance increase was promising, the clinical AI team discussed potential hurdles when implementing this CPM in practice. The major concern was that some of the CPM concepts were too vaguely defined, so that concept extractions for the same patient may differ between clinicians, reducing reliability in practice. So for Round 3, the AI agent was instructed to define concept questions more precisely using the prefix “Does the note mention the patient having ..., e.g.,....?” and likewise for surgery. For instance, a valid question could be “Does the note mention the patient having good exercise tolerance, e.g., having at least 5 METS of functional capacity or ability to climb two flights of stairs?” The clinician also suspected that home medications may also be predictive of AKI, so the AI agent was also encouraged to consider inclusion of medication keyphrases and concepts. The CPM in Round 3, the final round, achieved the highest AUC of 0.73 on the internal validation set (Period 1), suggesting that the increased precision reduced variability of the extracted concepts and thereby increased performance. Critically, the concepts now had construct validity since they were clearly defined. To assess temporal generalizability, the clinical AI team assembled a second test dataset from the disjoint time period of April-December 2024 (Period 2). The AUCs in Period 2 similarly improved from Round 1 in HACHI, starting from 0.61 in Round 1 to 0.77 and 0.76 in Rounds 2 and 3, respectively.

For comparison, we evaluated the unweighted and weighted Kheterpal models^45^ in two different ways: using LLM extractions from the preoperative clinical notes and using tabular data from the EHR. The models using LLM extractions achieved AUCs of 0.65–0.66 and those using tabular data achieved AUCs of 0.64. In addition, following our evaluation for TBI, we simulated a clinical expert who brainstormed predictors for AKI risk without an iterative refinement process by asking OpenEvidence^41^ to generate a list of potential predictors. Using the 30 concepts proposed through this approach, the resulting model achieved an AUC of 0.70. On the temporally disjoint test dataset, the Kheterpal models and the single-round brainstorming approach had similarly low AUCs of 0.67.

We additionally compare specificity values at a fixed sensitivity of approximately 0.90 (Table S4 shows these metrics broken down by time period). At this operating point, HACHI’s final-round CPM achieves a better sensitivity-specificity tradeoff than the baseline methods. For example, for Period 1, HACHI yields a sensitivity-specificity of 0.92–0.31, while the OpenEvidence baseline yields 0.91–0.27 and the Kheterpal baseline yields 1.00–0.00 (the closest achievable sensitivity to the target of 0.9 for the Kheterpal model).

## Discussion

This work demonstrates that meaningful collaboration between clinical AI teams and AI agents can produce high-performing CPMs that are simple to use, fully interpretable, and clinically credible. Across two clinical prediction tasks—traumatic brain injury in children and acute kidney injury in surgical patients—we found that the HACHI co-design framework consistently improved model performance, clinical relevance, and generalizability compared to existing clinical decision instruments and approaches that learn CPMs in a zero-shot fashion. Compared to the traditional process of training a CPM (which includes time spent on manual feature engineering, chart review, and iterative model refinement), HACHI constitutes a more efficient use of clinical AI teams: the teams in both case studies spent approximately 1–2 h reviewing results and providing feedback per round, with 3–4 rounds sufficient to reach a satisfactory model.

These case studies highlight how human oversight is essential not only for validating AI outputs but also for shaping the model development process in ways that the AI agent could not achieve in isolation. In the TBI case study, the clinical AI team identified critical issues that would have gone undetected in a purely automated pipeline: data leakage from transferred patients with prior CT results, spurious associations between documentation style and outcomes, and performance disparities across clinical sites with different patient populations and care pathways. These insights led to modifications of the AI-guided CPM learning procedure not only in terms of the prompts given (e.g., constraining concept definitions to patient characteristics rather than note features and asking the AI agent whether the concepts are expected to be risk factors versus protective factors) but also the data analyzed and how the model was evaluated (e.g., removing transferred patients and reweighting the training/validation data). Similarly, in the AKI case study, the AI agent initially focused exclusively on patient characteristics and learned concepts that were too vague for clinicians to implement in practice. The clinical AI team had to prompt the AI agent to additionally consider other concepts such as surgical factors and had to redefine how concept questions were formulated. Both case studies demonstrate how the clinical AI team must iteratively work with the AI agent to overcome the gulf of envisionment.

Through this collaborative process between the AI agent and clinical AI team, HACHI was able to uncover predictors previously overlooked in CPM developments, while recovering commonly used ones. In the TBI case study, the final model learned by HACHI is a much simpler form of the PECARN model, using only a subset of features such as LOC and altered mental status, alongside the more novel feature of “whether the patient has a normal gait.” In the AKI case study, the final model identified multiple factors that have not been commonly included in interpretable AKI risk scores: minimally invasive surgery, tachycardia, and sleep apnea. The more novel factors uncovered in this study may be useful more generally, although additional validation is necessary.

HACHI is designed to be simple to use, in light of recent concerns that a global CPM is unlikely to work well for all patient populations, given the complex nature of medicine^47^. Instead, these works suggest training local CPMs for local populations. To address this concern, HACHI has minimal requirements. It only needs access to (i) clinical notes, which are generally easy to extract from the EHR, (ii) a PHI-compliant LLM (e.g., API endpoints in the cloud), and (iii) guidance from a clinical AI team. Furthermore, the deployment of HACHI learned CPMs is straightforward. Rather than requiring LLMs in clinical practice, clinicians can instead annotate the questions during a patient encounter. The code for running HACHI is open-source and ready-to-use (see Code Availability section). This allows teams to run HACHI for local patient populations, where it can discover a parsimonious set of concepts that are highly predictive locally.

HACHI builds on recent work using LLMs for concept bottleneck models^24–27,48^ and iterative concept refinement^27–29,49^. These works stand in contrast to black-box methods for clinical text modeling (e.g., encoder-based modeling or AutoML^18,19,50,51^), which can achieve strong predictive performance but are not directly interpretable by clinicians, making them difficult to use, audit, and maintain. The most closely related method is BC-LLM^28^, which uses an LLM-in-the-loop rather than humans to iteratively propose and evaluate concepts. HACHI also relates to broader frameworks that combine LLMs with automatic verification loops^52–54^, that incorporate human feedback to improve machine learning models^55–59^ or that improve human-model interaction for difficult tasks^60–62^. Distinct from these prior works, HACHI integrates human domain expertise throughout the model development process, enabling clinical AI teams to identify data quality issues, enforce clinical consistency, and ensure practical usability—aspects that purely automated approaches cannot adequately address In this work, we have focused on linear CPMs for binary concepts, since the CPMs commonly used in clinical practice today fall in this category and binary concepts are both easier to extract and audit by clinicians. Furthermore, as illustrated in the Supplementary Information, we found in the TBI and AKI case studies that nonlinear CPMs did not outperform the simple models used in HACHI. Nevertheless, future work may extend HACHI to use more complex nonlinear models, e.g., sparse integer linear models^63^, Bayesian rule lists^64^, concept decision trees^65–67^, and others^5,68^.

The goal of HACHI is to close the gap from CPM development to clinical deployment. While HACHI makes significant strides in this direction, the learned CPM and its variables must still be thoroughly validated for the target deployment setting. This should include prospective testing and usability studies, as retrospective evaluation may not fully capture how models will perform when deployed prospectively in real-time clinical workflows. We emphasize that HACHI-derived CPMs have an advantage here, as the simplicity and interpretability of the learned models may facilitate more rapid prospective validation compared to complex black-box models. In addition, careful attention to fairness, bias, and equity is essential for understanding the model’s ability to generalize across patient populations and temporal shifts in the data^16,69–72^. To this end, the retrospective evaluations in this work across various strata provide an initial assessment of the temporal and cross-site generalizability of the learned CPMs. Future work may also consider using more sophisticated statistical tools that can identify which patient populations the model performs worst on^73^, which the AI agent can even use as feedback.

Additionally, while LLM-based concept extraction is scalable, it still requires careful prompt engineering and quality control. Moreover, the quality of HACHI-derived models depends on the LLM’s ability to accurately extract concepts from clinical notes and the types of clinical notes that are included. While prior work has found that GPT models are highly accurate at extracting concepts from medical texts^21,74^, LLMs differ in their extraction accuracy, which can affect the CPM’s performance. In our studies, we found that the iterative review process naturally led to more precise concept definitions. The clinical AI team’s feedback helped refine vague concepts like “protective factors” into precise questions that were more objective and had concrete examples, which substantially improved extraction consistency and is also likely to improve consistency across different LLMs.

A final set of limitations is that HACHI’s effectiveness can be limited by the expertise of the clinical AI team, a limitation common to all clinical data science pipelines. Future work should consider inter-team reproducibility when using and evaluating HACHI in sensitive prediction tasks. Nevertheless, we have provided a number of tools to improve the robustness of HACHI. First, a checklist of common failure modes is provided in the Supplementary Information (Table S6), which can aid clinical AI teams in knowing what to look for and provide feedback on. In addition, the Supplementary Information also includes templates for writing the initial prompts and adding feedback. Finally, we have tested HACHI’s sensitivity to prompt writing style and found that writing style had limited impact relative to inherent uncertainty (i.e., that due to limited sample sizes), as shown in the Supplementary Information.

Avenues for future research include extending HACHI’s applicability to more complex clinical scenarios by incorporating multimodal data such as imaging, laboratory results, or time series data, as has been done in prior CBM works^75,76^. In addition, other frameworks may be explored for incorporating human feedback into the co-design process, such as ways to reduce the burden on clinical experts while maintaining quality. Finally, although HACHI was tested in two clinical examples, the framework is applicable more broadly, particularly to fields beyond healthcare that also have large amounts of unstructured data. For this broader framework, perhaps a more suitable name is HACHII, “H uman+A gent C o-design for H ighly I nterpretable I nstruments.”

## Methods

### AI agentic CPM learning procedure

The AI agent is an encapsulated large language model (LLM) managed entirely through text-based prompts and outputs. The agent conducts a greedy hill-climbing procedure to select *k* concepts that are most predictive of the prediction target, subject to constraints specified by the clinical AI team. The procedure can be broken into the three main steps highlighted in Fig. 1a, beginning with the initialization in Step 1.

The first step of HACHI is to initialize a CPM, which will be the basis for concept refinement. To brainstorm possible concepts, HACHI iterates through the clinical notes in the dataset, appending each note to a KeyphrasePrompt. This prompt is used to instruct the LLM to extract a list of ‘keyphrases’ or ‘keywords’ that represent the content of the note. The data is then split into a training and validation partition. Applying a statistical tool (e.g., ridge-penalized logistic regression), HACHI fits a bag-of-words model on the training partition to identify keyphrases that are most associated with the outcome of interest. Next, to initialize the concepts, HACHI appends the top keyphrases to a ConceptInitializationProposalPrompt. This prompt instructs the LLM to make use of these keyphrases, together with its world knowledge, to propose *k* candidate “concept questions”. Concept questions are defined as human-interpretable yes/no questions, e.g., “Does this note mention the patient having a history of smoking?” Concept questions can encompass multiple keyphrases (i.e., the concepts may be hierarchical).

To convert the extracted concepts into a CPM, the *k* candidate “concept questions” are first extracted from the LLM’s response. For each question, HACHI iterates through the clinical notes, appending both the question and the note to a ConceptAnnotationPrompt, which instructs the LLM to give a yes/no response to the question posed regarding the note. Finally, HACHI fits a *k*-concept CPM on the training partition.

Now that the CPM has been initialized, HACHI cyclically traverses the concept positions *j* = 1, 2, …, *k*, evaluating each current concept for potential replacement by a more predictive concept. For each concept position, it conducts steps 2 and 3 as follows. In our experiments, we performed 10 iterations of the AI agent loop so that the concept in each position is refined at least once.

Step 2 of HACHI is to propose candidate concepts. The AI agent fits a bag-of-words model on the training partition to identify keyphrases that are most associated with the outcome of interest *Y*, but this time adjusting for existing concepts that are not up for replacement. HACHI appends the top keyphrases to a ConceptReplacementProposalPrompt, which instructs the LLM to make use of these keyphrases, together with its world knowledge, to propose *m* candidate replacement “concept questions”. The *m* candidate “concept questions” are extracted from the LLM’s response.

Step 3 of HACHI is to evaluate candidate concepts. For each question, the AI agent iterates through the clinical notes, appending both the question and the note to a ConceptAnnotationPrompt, which instructs the LLM to give a yes/no response to the question posed regarding the note. The resulting annotations are then used by the AI agent to fit a *k*-concept CPM (again using lasso-penalized logistic regression via a python tool call) on the training partition for each candidate concept by combining the candidate with the existing *k* − 1 concepts. Then, HACHI evaluates the resulting CPM’s performance on the validation partition by applying a statistical validation metric via (e.g., AUC), again using a python tool call. If the best-performing candidate concept outperforms the existing concept being considered for replacement, HACHI replaces it with this best-performing candidate.

The AI-guided CPM learning procedure is stochastic, due to both sample splitting and inclusion of the AI agent. Furthermore, there is inherent uncertainty regarding the true concepts that are relevant for predicting the outcome of interest, due to finite sample sizes and the infinite number of possible concepts. To address both concerns, we run the AI-guided CPM learning procedure for multiple seeds in parallel. Differences between the learned CPMs can reveal uncertainty in the truly relevant concepts and stability of the learning process, enabling more efficient Human-AI interaction. See the Supplementary Information for HACHI results from other seeds.

### Optimizing HACHI for human-AI co-design

The key contribution of HACHI is the formalization of previously ad-hoc workflows into a more standardized process for enabling human-AI co-design. This requires careful thought, since one cannot simply combine any AI-based CPM learning procedure and ask human experts to provide input. Rather, from a Human-Computer Interaction (HCI) perspective, there are three key requirements to ensure a truly collaborative and effective human+AI co-design process:

1. *Transparency*: The clinical AI team should be able to fully interpret the AI agent’s learning process and the resulting CPM, as it is necessary for the clinical AI team to generate relevant feedback.
2. *Steerability*: The AI agent in the CPM learning procedure is amenable to steering by a clinical AI team.
3. *Reciprocal learning*: In addition to the AI agent being able to learn from the clinical AI team, the clinical AI team should also be able to learn from the AI agent.

Below, we discuss how HACHI was designed to satisfy and optimize each of these axes.

HACHI provides *transparency* through two key mechanisms. First, the AI agent-based CPM learning procedure in HACHI provides explanations at every step, including explanations which concepts it is exploring, how results from statistical modeling guided it towards that direction, and how it assessed candidate concepts for clinical validity. Furthermore, unlike many recent works using AI to learn concept bottleneck models, the concepts used in HACHI are fully interpretable because they are binary yes-no questions ("hard concepts”), rather than pseudo-concepts such as those based on embeddings (also known as “soft concepts”)^28^. Second, to help the human expert determine how the prompts should be modified each round, an interactive user interface was built so that clinical AI teams can scrutinize the AI agent’s reasoning process and accuracy. In particular, a PHI-compliant single-page locally-hosted webpage was built to show: (i) the learned factors, (ii) the LLM concept annotations at a patient/note level, and (iii) the performance of the CPM on a held-out test set; see Fig. 2 for more details. Furthermore, this interface prioritizes cases where the AI agent was incorrect so that experts can be most efficient in providing feedback.

HACHI provides *steerability* of the AI agent primarily through four key points of access, corresponding to prompts: KeyphrasePrompt, ConceptInitializationProposalPrompt, ConceptReplacementProposalPrompt, and ConceptAnnotationPrompt. In the Supplementary Information, we provide general templates for both writing the initial prompts and how feedback can be incorporated into these prompts. When needed, teams may also opt for other ways to steer the AI agent as well, such as adding additional directions for data preprocessing, modifying the statistical tools used by the AI agent, and restricting which candidate concepts can be selected in the optimization process. More complex modifications may require code-based changes to the AI-guided CPM learning procedure, rather than simply modifying prompts. To assist clinical AI teams in deciding how to steer the AI agent, the Supplementary Information includes a basic checklist (Table S6) for reviewing results from the AI agent and giving it feedback.

As illustrated in the case studies, HACHI is also designed to facilitate *reciprocal learning*, wherein the AI agent does not just learn from the clinical AI team but also in the reverse direction. Reciprocal learning requires not only transparency and steerability, but also the ability of the AI agent to derive insights that the clinical AI team can learn from and improve on. As such, the AI agent in HACHI was based on BC-LLM^28^, which is unique in its ability to discover *new* concepts from the data and does not just limit itself to those initially suggested by the clinical AI team. To further optimize reciprocal learning, we simplified BC-LLM and made it more computationally efficient—running 2–3 times as fast as the original method—to have shorter feedback cycles. This was critical to ensure full engagement, and thus reciprocal learning, by the clinical AI team. Finally, we found it was critical to have interdisciplinary meetings to fully enable reciprocal learning, as data scientists were helpful in helping translate what the AI agent was learning to other team members, and vice versa.

### Data collection for traumatic brain injury (TBI)

The dataset was assembled across two academic pediatric EDs within the Benioff Children’s Hospital (Oakland and Mission Bay Campuses). Data is fully de-identified and is not considered human subjects research by the Institutional Review Board at the University of California, San Francisco. To ensure a comprehensive capture of relevant cases, we included any encounter with documented head trauma or a traumatic brain injury (TBI) diagnosis, as coded by the following ICD-10 (International Classification of Diseases, Tenth Revision [ICD-10]) codes: S06, S00.03, S00.83, S00.93, S02.0, S02.1, S02.80, S02.81, S02.91, S07.1, S07.8, S07.9, S09.8, and S09.90 for head trauma, and S06.1, S06.2, S06.3, S06.5, S06.6, S06.89, S06.9, S04.02, S04.03, S04.04, S07.1, and T74.4 for TBI. Encounters with at least one qualifying hospital-wide diagnostic code within the same encounter or occurring up to one calendar day before or after the ED visit were included, to capture both ED and Inpatient Diagnoses. Concatenated, the notes per encounter contain an average of 1454 tokens. Table 3 (top) shows the prevalence of various concepts in the final dataset.

**Table 3:** Final concepts in CPM for AKI and TBI learned by HACHI

TBI Concept	Coefficient	Prevalence
... the patient having normal gait?	−0.22	0.56 (0.51, 0.61)
... the patient experiencing head trauma?	0.08	0.95 (0.93, 0.97)
... the patient experiencing a headache?	0.25	0.42 (0.37, 0.47)
... the patient having altered mental status?	0.73	0.13 (0.10, 0.16)
... the patient experiencing unconsciousness?	1.58	0.39 (0.34, 0.44)

AKI Concept	Coefficient	Prevalence
... the surgery being minimally invasive, e.g., laparoscopic or robotic-assisted procedures?	−0.74	0.41 (0.37, 0.44)
... the patient having hypertension, e.g., high blood pressure or antihypertensive medication use?	0.31	0.54 (0.51, 0.58)
... the patient having obesity, e.g., high BMI or overweight status?	0.39	0.32 (0.29, 0.36)
... the surgery being urgent or emergent, e.g., requiring immediate attention or intervention?	0.50	0.21 (0.18, 0.23)
... the patient having a history of sleep apnea, e.g., witnessed apnea or CPAP use?	0.51	0.20 (0.17, 0.23)
... the patient having heart failure, e.g., reduced ejection fraction or history of heart failure exacerbations?	0.64	0.08 (0.06, 0.10)
... the patient having an active systemic infection, e.g., sepsis, bacteremia, or requiring therapeutic antibiotics?	0.72	0.07 (0.05, 0.09)
... the patient having tachycardia, e.g., heart rate >100 bpm or palpitations?	0.88	0.16 (0.14, 0.19)
... the surgery being major or high-risk, e.g., major abdominal surgery or anticipated blood loss > 500 mL?	0.91	0.40 (0.36, 0.43)
... the patient having chronic kidney disease, e.g., a history of CKD or elevated creatinine levels?	1.05	0.13 (0.11, 0.16)

Coefficients and prevalences shown.

We note that that the Oakland campus operates as a tertiary referral center, so a substantial portion of these patients are referrals/transfers. Nevertheless, not all of these patients have prior diagnoses and/or imaging results, so the team intentionally avoided excluding all transferred patients and an iterative auditing process was used instead to filter for the clinically relevant population.

### Data collection for acute kidney injury (AKI)

The retrospective case-control cohort was assembled from all General Surgery cases between January 2016 and March 2024 at UCSF. The study protocol and data collection were reviewed and approved by the Institutional Review Board at the University of California, San Francisco (Protocol #17-23204). Inclusion criteria included adult patients undergoing inpatient procedures (including emergency surgeries) with at least one serum creatinine (sCr) value in the 90 days preceding surgery and at least one serum sCr in the 7 days following surgery. The main outcome was AKI according to the KDIGO criteria in the 7 days following surgery: [48 h maximum postoperative sCr] - [last preoperative sCr] ≥ 0.3 mg/dL or [7 day maximum postoperative sCr]/[last preoperative sCr] ≥ 1.5^46^. We analyze the preoperative note written by the anesthesiologist, which has an average of 1991 tokens. Table 3 (bottom) shows the prevalence of various concepts in the final dataset. Among the AKI cases, the prevalence of AKI Stage 1 was 0.74 (0.66, 0.80), AKI Stage 2 was 0.12 (0.08, 0.14), and AKI Stage 3 was 0.16 (0.12, 0.20).

## Supplementary information

Supplementary Information

## Data Availability

Data that support the findings of this study are not publicly available due to the use of clinical notes, which constitute protected health information. Synthetic data for testing HACHI is available in the associated open-source software package on Github.An open-source python package for the HACHI framework is publicly available at http://github.com/jjfenglab/HACHI, which includes code for reproducing this work, web interfaces for reviewing outputs from the AI agent, and tutorials for running the AI-guided CPM learning procedure.

## Code availability

An open-source python package for the HACHI framework is publicly available at http://github.com/jjfenglab/HACHI, which includes code for reproducing this work, web interfaces for reviewing outputs from the AI agent, and tutorials for running the AI-guided CPM learning procedure.
