# Supplementary material for: Human-AI co-design for clinical prediction models
Source: NPJ Digit Med. 2026 Jun 6;9:798. doi: 10.1038/s41746-026-02838-5 (PMC13669369; doi:10.1038/s41746-026-02838-5)
Supplement: Supplementary file 1 [file 41746_2026_2838_MOESM1_ESM.pdf]

# A Supplementary Information

**Table S1: Complete list of concepts for TBI prediction across HACHI iterations.** In round 1, we additionally consider running HACHI in an outcome-agnostic way, where the LLM does not know which outcome it is predicting and must rely on the prediction model to identify useful features. Nevertheless, the model successfully identifies useful features, even recovering a few of the original PECARN concepts.

| **Round** | **Coef.** | **Concepts** |
|---|---|---|
| **Round 1** | 2.70 | Does the note mention the patient experiencing a loss of consciousness? |
| | 1.74 | Does the note mention a brain bleed? |
| | 1.58 | Does the note mention the patient having a neurological event? |
| | 1.23 | Does the note mention a Glasgow Coma Scale score? |
| | 1.10 | Does the note mention the patient being seizure-free? |
| **Round 2** | 3.46 | Does the note mention the patient experiencing loss of consciousness? |
| | 1.50 | Does the note mention the patient having a normal Glasgow Coma Scale score and being at least 2 years old? |
| | 1.04 | Does the note mention the patient experiencing convulsions? |
| | 0.82 | Does the note mention the patient having altered mental status? |
| | -0.65 | Does the note mention the patient having intact cranial nerves? |
| **Round 3** | 4.34 | Does the note mention the patient experiencing loss of consciousness? |
| | 1.41 | Does the note mention the patient having a history of mild TBI? |
| | 1.21 | Does the note mention the patient having an occipital hematoma? |
| | 1.07 | Does the note mention the patient having vision changes? |
| | -0.53 | Does the note mention the patient having memory intact? |
| **Round 4** | 1.58 | Does the note mention the patient experiencing unconsciousness? |
| | 0.73 | Does the note mention the patient having altered mental status? |
| | 0.25 | Does the note mention the patient experiencing a headache? |
| | 0.08 | Does the note mention the patient experiencing head trauma? |
| | -0.22 | Does the note mention the patient having normal gait? |

**Table S2: Comparing the sensitivity and specificity of TBI models from HACHI and comparator methods.** To make the models comparable, the threshold is chosen so that the sensitivity is as close to 0.90 as possible. 95% CI is shown in parentheses.

| Model | Baseline (PECARN) | OpenEvidence+ LLMs | HACHI (Round 1) | HACHI (Round 2) | HACHI (Round 3) | HACHI (Round 4) |
|---|---|---|---|---|---|---|
| Sensitivity | 0.942 (0.942, 1.000) | 0.913 (0.900, 1.000) | 0.906 (0.900, 0.993) | 0.913 (0.900, 0.938) | 0.906 (0.900, 0.950) | 0.906 (0.900, 0.938) |
| Specificity | 0.556 (0.000, 0.613) | 0.642 (0.000, 0.884) | 0.481 (0.154, 0.906) | 0.615 (0.510, 0.875) | 0.749 (0.332, 0.891) | 0.807 (0.545, 0.883) |

**Table S3: Complete list of concepts for AKI prediction across HACHI iterations.**

| Round | Coef. | Concepts |
|---|---|---|
| **1** | 1.31 | Does the note mention this patient having leukocytosis? |
| | 0.87 | Does the note mention this patient having abdominal distention? |
| | 0.74 | Does the note mention this patient having cardiac dysfunction? |
| | 0.71 | Does the note mention this patient having tachycardia? |
| | 0.65 | Does the note mention this patient experiencing swelling? |
| | 0.57 | Does the note mention this patient having a systemic infection? |
| | 0.53 | Does the note mention this patient having renal impairment? |
| | 0.47 | Does the note mention this patient having diabetes mellitus? |
| | -0.36 | Does the note mention this patient having no kidney disease? |
| | -1.01 | Does the note mention this patient having low hematocrit? |
| **2** | 1.28 | Does the note mention the patient having chronic kidney disease? |
| | 1.03 | Does the note mention the patient undergoing an exploratory laparotomy? |
| | 0.98 | Does the note mention the patient having sepsis? |
| | 0.89 | Does the note mention the patient having fluid retention? |
| | 0.47 | Does the note mention the patient having a respiratory condition? |
| | 0.43 | Does the note mention the patient requiring a blood transfusion during surgery? |
| | 0.40 | Does the note mention the surgery being high-risk? |
| | 0.37 | Does the note mention the patient having heart disease? |
| | 0.31 | Does the note mention the patient having a malignancy? |
| | -1.01 | Does the note mention the patient having minimally invasive surgery? |
| **3** | 1.05 | Does the note mention the patient having chronic kidney disease, e.g., a history of CKD or elevated creatinine levels? |
| | 0.91 | Does the note mention the surgery being major or high-risk, e.g., major abdominal surgery or anticipated blood loss > 500mL? |
| | 0.88 | Does the note mention the patient having tachycardia, e.g., heart rate > 100 bpm or palpitations? |
| | 0.72 | Does the note mention the patient having an active systemic infection, e.g., sepsis, bacteremia, or requiring therapeutic antibiotics? |
| | 0.64 | Does the note mention the patient having heart failure, e.g., reduced ejection fraction or history of heart failure exacerbations? |
| | 0.51 | Does the note mention the patient having a history of sleep apnea, e.g., witnessed apnea or CPAP use? |
| | 0.50 | Does the note mention the surgery being urgent or emergent, e.g., requiring immediate attention or intervention? |
| | 0.39 | Does the note mention the patient having obesity, e.g., high BMI or overweight status? |
| | 0.31 | Does the note mention the patient having hypertension, e.g., high blood pressure or antihypertensive medication use? |
| | -0.74 | Does the note mention the surgery being minimally invasive, e.g., laparoscopic or robotic-assisted procedures? |

**Table S4: Comparing the sensitivity and specificity of AKI models from HACHI and comparator methods.** To make the models comparable, the threshold is chosen so that the sensitivity is as close to 0.90 as possible. 95% CI is shown in parentheses.

| Model | Baseline (Kheterpal) | OpenEvidence+ LLMs | HACHI (Round 1) | HACHI (Round 2) | HACHI (Round 3) |
|---|---|---|---|---|---|
| Sensitivity in Domain 1 | 1.000 (0.703, 1.000) | 0.910 (0.900, 0.942) | 0.902 (0.900, 0.929) | 0.902 (0.900, 0.945) | 0.915 (0.900, 0.925) |
| Specificity in Domain 1 | 0.000 (0.000, 0.629) | 0.274 (0.173, 0.331) | 0.097 (0.050, 0.124) | 0.249 (0.139, 0.328) | 0.312 (0.209, 0.429) |
| Sensitivity in Period 1 | 1.000 (1.000, 1.000) | 0.915 (0.900, 0.965) | 0.915 (0.900, 0.962) | 0.932 (0.903, 0.986) | 0.932 (0.900, 0.965) |
| Specificity in Period 2 | 0.000 (0.000, 0.000) | 0.288 (0.125, 0.483) | 0.169 (0.019, 0.415) | 0.441 (0.286, 0.579) | 0.373 (0.220, 0.677) |

# B Variability and Sensitivity of HACHI

When running HACHI, the AI agentic CPM learning procedure is run for two random seeds to provide uncertainty quantification of the learned concepts; concepts that show up consistently across multiple seeds have higher certainty while concepts that only appear in one seed have lower certainty. This uncertainty stems in large part due to inherent randomness due to limited sample size, also known as

aleatoric uncertainty [28]. The final results presented in the main manuscript use the best seed, selected based on its test AUC. In addition, to understand the sensitivity of HACHI to prompt construction, we include the learned concepts and test AUCs when the prompts for the last round of the TBI and AKI case studies are rewritten. Table S5 shows results from the alternative seed and the perturbed prompts for the two case studies. We note that variability in the learned model due to prompt writing style is not evidently larger than that due to random seeds, so prompt writing style is not the major driver of stochasticity in the outputs.

**Table S5**: Evaluating variability of learned concepts, odds ratios, and test AUCs of CPMs by HACHI across different random seeds and prompt rephrasings. Results are shown for the TBI and AKI case studies.

| Prompt | Odds Ratio | Concepts | AUC |
|---|---|---|---|
| **TBI: Different seed** | 28.514 | Does the note mention the patient experiencing loss of consciousness? | 0.915 (0.882, 0.944) |
| | 3.673 | Does the note mention the patient having an occipital hematoma? | |
| | 2.987 | Does the note mention the patient having a frontal headache? | |
| | 1.035 | Does the note mention the patient having a history of concussion? | |
| | 0.571 | Does the note mention the patient being alert and responsive? | |
| **TBI: Rephrased final-round prompt** | 5.092 | Does the note mention the patient experiencing loss of consciousness? | 0.912 (0.880, 0.945) |
| | 2.063 | Does the note mention the patient having altered mental status? | |
| | 1.286 | Does the note mention the patient experiencing a headache? | |
| | 1.182 | Does the note mention the patient having a hematoma on the head? | |
| | 0.877 | Does the note mention the patient experiencing vertigo? | |
| **AKI: Different seed** | 0.701 | Does the note mention the patient having good exercise tolerance, e.g., having at least 5 METS of functional capacity or ability to climb two flights of stairs? | 0.696 (0.658, 0.733) |
| | 0.704 | Does the note mention the patient having normal pulmonary function, e.g., clear lungs or normal breath sounds? | |
| | 1.205 | Does the note mention the patient being elderly, e.g., age over 65? | |
| | 1.510 | Does the note mention the patient having high BMI, e.g., BMI over 30 indicating obesity? | |
| | 1.530 | Does the note mention the patient having abnormal pulmonary function, e.g., shortness of breath, wheezing, or use of home respiratory treatments? | |
| | 1.593 | Does the note mention the patient having a high risk of bleeding, e.g., coagulopathy, bleeding diathesis, or need for blood transfusion? | |
| | 1.731 | Does the note mention the patient having loose teeth or other dental problems, e.g., dental caries or periodontal disease? | |
| | 2.027 | Does the note mention the surgery being an emergency or urgent procedure? | |
| | 2.170 | Does the note mention the patient having an active systemic infection, e.g., sepsis or bacteremia? | |
| | 2.573 | Does the note mention the patient undergoing major abdominal surgery, e.g., open abdominal aortic aneurysm repair or Whipple procedure? | |
| **AKI: Rephrased final-round prompt** | 0.536 | Does the note mention the surgery being minimally invasive, e.g., laparoscopic or robotic surgery? | 0.735 (0.699, 0.768) |
| | 0.648 | Does the note mention the patient having good exercise tolerance, e.g., having at least 5 METS of functional capacity or ability to climb two flights of stairs? | |
| | 0.718 | Does the note mention the patient having normal pulmonary function, e.g., clear lungs or normal breath sounds? | |
| | 1.010 | Does the note mention the patient having elevated blood pressure, e.g., hypertension or high blood pressure? | |
| | 1.150 | Does the note mention the patient being male, e.g., male sex or gender? | |
| | 1.204 | Does the note mention the patient having cardiovascular risk factors, e.g., history of heart disease or high cardiovascular risk? | |
| | 1.364 | Does the note mention the surgery being urgent or emergent, e.g., urgent operation or emergent surgery? | |
| | 1.405 | Does the note mention the patient having a systemic infection, e.g., sepsis or bacteremia? | |
| | 1.670 | Does the note mention the patient having chronic kidney disease, e.g., a history of CKD or elevated creatinine levels? | |
| | 1.747 | Does the note mention the patient undergoing major abdominal surgery, e.g., exploratory laparotomy or major abdominal procedures? | |

# C Checklist for Clinical AI teams

The TBI and AKI case studies in this work revealed that clinical AI teams can and should engage with the entire AI-based CPM learning procedure, from data pre-processing to evaluation. Although the range of potential feedback can be overwhelming, this process shares many similarities with traditional CPM development. Thus, building on findings in this work as well as existing guidelines for developing and evaluating CPMs [77, 78], we provide a checklist to guide clinical AI teams in reviewing AI agent outputs. Clinical AI teams can use this checklist to systematically evaluate the AI outputs and improve their agentic pipeline.

**Table S6**: Checklist when reviewing HACHI outputs in each round

| Category | Example assessment questions | Example improvements |
|---|---|---|
| **Data** | Does the data include the relevant signals? for prediction? | Add more data sources and note types |
| | Does the data reflect the target population? | Modify inclusion/exclusion criteria, identify potential data quality issues from learned concepts, add (AI-based) filtering step |
| | Was the appropriate data used? | Identify potential data quality issues from learned concepts, add missing data sources if needed |
| **Concepts/ Variables** | Are the concepts objectively defined? | Encourage the AI agent to explore objective, measurable concepts |
| | Are concept extractions well-aligned with human extractions? | Use a more powerful AI model, improve AI context management, require AI agent to refine concept questions for clarity, encourage the AI agent to explore concepts that are to be more objective |
| | Are learned concepts available at the intended time of use? | Modify inclusion/exclusion criteria, add (AI-based) filtering step, provide guidance on keyphrase extraction and concept exploration |
| | Is there significant variability in the learned concepts across seeds? | Refine prompts to provide specific guidance on which concept categories to prioritize, collect additional data to provide more signals for relevant concepts, run additional seeds to assess consistency |
| | Do concepts make clinical sense? | Review annotated concepts and clinical notes to an expert clinician through a UI, provide guidance to the AI agent to explore additional concepts suggested by the clinician |
| | Would the end user of this CPM be able to extract these concepts? | Integrate expert clinical knowledge to identify the feasibility of extracting these concepts during clinical practice, restrict the AI agent to explore concepts that are specific and can be reliably annotated during clinical practice |
| | Are there concept categories that the AI agent missed or failed to consider? | Compare the learned CPM against baseline CPMs, review results with an expert clinician to identify missing concept categories, guide the AI agent toward unexplored relevant categories |
| | Are concept coefficients aligned with clinical intuition? | Constrain the AI agent to select concepts whose coefficient aligns with clinical priors, identify and mitigate causes of spurious correlations |
| **Outcome** | Were outcomes defined and assessed appropriately? | Confirm outcome definitions align with clinical standards and the model's intended use and update the outcome extraction definition if needed, refine inclusion/exclusion criteria to match the outcome definition |
| | Is outcome information being leaked into the concepts? | Provide guardrails to the AI agent to explore concepts that do not include outcome information, update data inclusion/exclusion criteria to remove cases that lead to the data leakage |
| **Training/ Evaluation** | Does the model performance significantly differ between subgroups or timespans? | Identify root causes of performance disparities, collect additional data from under performing subgroups or modify the training objective to encourage more uniform performance |

## D Additional experiments

**Tradeoff between simple and complex models.** The choice of binary concepts and a linear model represents a deliberate tradeoff between interpretable/auditable performance and black-box peak performance. Binary concepts are reliably extractable by LLMs and directly auditable by clinicians, while linear models allow transparent inspection of each concept's contribution. However, one may be concerned that this representational choice may incur a drop in predictive performance in some settings. To assess this restriction, we replaced the linear model used in the OpenEvidence baseline with a random forest (fit using default scikit-learn hyperparameters [79]). For TBI, this yields a small AUC improvement (0.871 versus the original 0.850), but for AKI it did not yield any performance improvement (in fact it slightly hurt performance). Nevertheless, in both case studies, we find that the use of a nonlinear model alone does not outperform linear HACHI-derived models, illustrating how jointly searching over an infinite concept space while learning a linear model can in fact outperform using a nonlinear model for a fixed set of concepts. Future work may also consider extending HACHI to use nonlinear prediction models as well as non-binary concepts.

# E Prompts: TBI

Here we show examples of prompts used for TBI in Round 1; see all prompts on Github.

## E.1 Round 1

### E.1.1 KeyphrasePrompt

```
You are extracting clinical descriptors of a pediatric patient from emergency room medical notes that may influence
↪  their risk of being diagnosed with traumatic brain injury (TBI).  Focus entirely on factors explicitly mentioned
↪  in the notes. Do not infer or add details unless explicitly stated.

Extract factors that may:
- Increase TBI risk, be specific and detailed (e.g., substance use, prior head injuries)
- Decrease TBI risk, be specific and detailed (e.g., protective equipment use, medication compliance)
- Indicate TBI severity or complications, be specific and detailed (e.g., neurological symptoms, cognitive changes)

For each factor, provide:
1. The primary descriptor (exact or paraphrased from the note)
2. Synonyms or alternative phrasings
3. Broader generalizations that capture similar concepts

Requirements:
- Each descriptor should be $\leq$3 words
- The descriptor should state distinguishing features.
- List specific terms, synonyms, and increasingly general concepts. However, do not state overly general concepts like
↪  "protective factor", "risk factor", "low risk", or "high risk" because we are using these terms to
↪  brainstorm/learn specific factors that clinicians can use to predict TBI risk.
- Separate items with commas


Note(s):
"{note}"

Do not infer or fabricate details. Base all responses strictly on the medical note text.

Output Format:
{
    "reasoning": "Describe any reasoning here",
    "keyphrases": [
        "Primary descriptor, Synonym1, Synonym2, Broader category",
        "Primary descriptor, Alternative phrasing, More general concept",
        ...
    ]
}

Examples:
{
    "reasoning": "Describe any reasoning here",
    "keyphrases": [
        "Crowded residence, Overcrowded living, Constrained environment, Environmental risk",
        "Cervical strain, Neck pain, Muscle spasm",
        "Physical assault, Blunt trauma, Injury infliction",
        ...
    ]
}
```

### E.1.2 ConceptInitializationProposalPrompt

```
We are fitting a concept bottleneck model to predict traumatic brain injury (TBI) for pediatric patients admitted to
↪  emergency room. The goal is to come up with a few meta-concepts that clinicians can use to easily risk-stratify
↪  patients for whether they are at high risk of being diagnosed with TBI. As training data, we are using clinical
↪  notes to retrospectively extract patient characteristics.

The top words most associated with TBI were estimated using a simple logistic regression model with tf-idf features.
↪  Based on the top words, come up with {max_meta_concepts} meta-concepts. A meta-concept has to be defined in terms
↪  of a yes/no question, e.g. "Does the note mention this patient having a fall?". Pick meta-concepts that are as
↪  specific as possible, without being too broad.
```

```
Suggestions for generating candidate meta-concepts: Do not propose meta-concepts that are simply a union of two different concepts (e.g. "Does the note mention this patient having an infection or being elderly?" is not allowed), questions with answers that are almost always a yes (e.g. the answer to "Does the note mention this patient being hospitalized?" is almost always yes), or questions where the yes/no options are not clearly defined (e.g. "Does the note mention this patient experiencing difficulty?" is not clearly defined because difficulty may mean financial difficulty, physical difficulties, etc).

Words with top coefficients:

{top_features_df}

Propose at least {max_meta_concepts} candidates in the following JSON format:
{
  "reasoning": "Step-by-step explanation of how you systematically worked through the model concepts (from most to least predictive).",
  "concepts": [
    {
      "concept": "<QUESTION 1>",
      "words": ["<SYNONYM/ANTONYM 1>", "<SYNONYM/ANTONYM 2>", "<SYNONYM/ANTONYM 3>"]
    },
    {
      "concept": "<QUESTION 2>",
      "words": ["<SYNONYM/ANTONYM 1>", "<SYNONYM/ANTONYM 2>", "<SYNONYM/ANTONYM 3>"]
    },
    ...
    {
      "concept": "<QUESTION {max_meta_concepts}>",
      "words": ["<SYNONYM/ANTONYM 1>", "<SYNONYM/ANTONYM 2>", "<SYNONYM/ANTONYM 3>"]
    }
  ]
}

Example answer:
{
  "reasoning": "Step-by-step reasoning through the model concepts",
  "concepts": [
    {
      "concept": "Does the note mention the patient experiencing head trauma?",
      "words": ["head injury", "skull trauma", "cranial damage"]
    },
    {
      "concept": \Does the note mention loss of consciousness?"
      "words": ["LOC", "fainting, unresponsive"]
    },
    ...
    ]
}
```

### E.1.3 ConceptReplacementProposalPrompt

```
The goal is to come up with a concept bottleneck model (CBM) that extracts {num_concepts} meta-concepts from clinical notes from the electronic health record of pediatric patients admitted to the emergency room to predict risk of having a traumatic brain injury (TBI). A meta-concept is a binary feature extractor defined by a yes/no question. We have {num_concepts_fixed} meta-concepts so far:
{meta_concepts}

To come up with the {num_concepts}th meta-concept, I have done the following: I first fit a CBM on the {num_concepts_fixed} existing meta-concepts. Then to figure out how to improve this {num_concepts_fixed}-concept CBM, I first extracted a list of concepts that are present in each note, and then fit a linear regression model on the extracted concepts to predict the residuals of the {num_concepts_fixed}-concept CBM. These are the top extracted concepts in the resulting residual model, in descending order of importance:

{top_features_df}

To interpret this residual model, a general rule of thumb is that an extracted concept with a large positive coefficient means that a high risk of TBI is positively associated with the concept being mentioned in the note, and an extracted concept with a negative coefficient means that a higher risk of TBI is negatively associated with the concept being mentioned in the note.
```

```
Given the residual model, create cohesive candidates for the {num_concepts}th meta-concept. Be systematic and consider all the listed concepts in the residual model. Start from the most to the least predictive concept. For each concept, check if it matches an existing meta-concept or create a new candidate meta-concept. Work down the list, iterating through each concept. Clearly state each candidate meta-concept as a yes/no question.

Suggestions for generating candidate meta-concepts: Do not propose meta-concepts that are simply a union of two different concepts (e.g. "Does the note mention this patient having an infection or being elderly?" is not allowed), questions with answers that are almost always a yes (e.g. the answer to "Does the note mention this patient being hospitalized?" is almost always yes), or questions where the yes/no options are not clearly defined (e.g. "Does the note mention this patient experiencing difficulty?" is not clearly defined because difficulty may mean financial difficulty, physical difficulties, etc). Do not propose meta-concepts where you would expect over 95% agreement or disagreement with the {num_concepts_fixed} existing meta-concepts (e.g. "Does the note mention the patient having homelessness?" overlaps too much with "Does the note mention the patient having housing insecurity?").

Finally, summarize all the generated candidates for the {num_concepts}-th meta-concept in a JSON. Merge any candidate meta-concepts that are essentially the same (where you would expect over 95% agreement) or essentially opposites (you would expect over 95% disagreement). In the JSON, include a list of comma-separated list of phrases that mean the same or opposite of each candidate meta-concept. Propose at least ten candidates. The final JSON should have the following format:

Propose at least six candidates in the following JSON format:
{
  "reasoning": "Step-by-step explanation of how you systematically worked through the model concepts (from most to least predictive) and combined them with the feedback suggestions.",
  "concepts": [
    {
      "concept": "<QUESTION 1>",
      "words": ["<SYNONYM/ANTONYM 1>", "<SYNONYM/ANTONYM 2>", "<SYNONYM/ANTONYM 3>"]
    },
    {
      "concept": "<QUESTION 2>",
      "words": ["<SYNONYM/ANTONYM 1>", "<SYNONYM/ANTONYM 2>", "<SYNONYM/ANTONYM 3>"]
    },
    ...
    {
      "concept": "<QUESTION 6>",
      "words": ["<SYNONYM/ANTONYM 1>", "<SYNONYM/ANTONYM 2>", "<SYNONYM/ANTONYM 3>"]
    }
  ]
}

Example answer:
{
  "reasoning": "Step-by-step reasoning through the residual model concepts and feedback",
  "concepts": [
    {
      "concept": "Does the note mention the patient experiencing head trauma?",
      "words": ["head injury", "skull trauma", "cranial damage"]
    },
    {
      "concept": \Does the note loss of consciousness?"
      "words": ["LOC", "fainting, unresponsive"]
    },
    ...
    ]
}
```

### E.1.4 ConceptAnnotationPrompt

```
You will be given clinical notes. I will give you a series of questions. Your task is answer each question with a probability from 0 to 1. Summarize the response with a JSON that includes your answer to all of the questions. Questions:
{prompt_questions}

clinical notes:
{sentence}

Example answer: {
    "0": 0,
    "1": 1,
    "2": 0.5
```

```
}
Answer all the questions and do not answer with anything else besides valid JSON. Do not add comments to the JSON.
```

## E.2 Round 2

### E.2.1 KeyphrasePrompt

```
You are extracting clinical descriptors of a pediatric patient from emergency room medical notes that may influence their risk of being diagnosed with traumatic brain injury (TBI).  Focus entirely on factors explicitly mentioned in the notes. Do not infer or add details unless explicitly stated.

Extract factors that may:
- Increase TBI risk, be specific and detailed (e.g., substance use, prior head injuries, loss of consciousness, cognitive delays, history of traumatic brain injury, vomiting, headaches, hematoma, location of hematoma, GCS<15)
- Decrease TBI risk, be specific and detailed (e.g., protective equipment use, medication compliance, GCS=15)
- Indicate TBI severity or complications, be specific and detailed (e.g., seizures, cognitive changes, somnolence)

For each factor, provide:
1. The primary descriptor (exact or paraphrased from the note)
2. Synonyms or alternative phrasings
3. Broader generalizations that capture similar concepts

Requirements:
- Each descriptor should be $\leq$3 words
- The descriptor should state distinguishing features. Focus on clinically relevant factors.
- List specific terms, synonyms, and increasingly general concepts. However, do not state overly general concepts like "protective factor", "risk factor", "low risk", or "high risk" because we are using these terms to brainstorm/learn specific factors that clinicians can use to predict TBI risk.
- Do not extract concepts that are sensitive to the hospital's operational protocols. So rather than extracting concepts that simply describe patients having a neurological assessment, propose concepts that are characteristics or symptoms of the patient themself.
- Separate items with commas

Note(s):
"{note}"

Do not infer or fabricate details. Base all responses strictly on the medical note text.

Output Format:
{
    "reasoning": "Describe any reasoning here",
    "keyphrases": [
        "Primary descriptor, Synonym1, Synonym2, Broader category",
        "Primary descriptor, Alternative phrasing, More general concept",
        ...
    ]
}

Examples:
{
    "reasoning": "Describe any reasoning here",
    "keyphrases": [
        "Crowded residence, Overcrowded living, Constrained environment, Environmental risk",
        "Cervical strain, Neck pain, Muscle spasm",
        "Physical assault, Blunt trauma, Injury infliction",
        ...
    ]
}
```

### E.2.2 ConceptInitializationProposalPrompt

```
We are fitting a concept bottleneck model to predict traumatic brain injury (TBI) for pediatric patients admitted to emergency room. The goal is to come up with a few meta-concepts that clinicians can use to easily risk-stratify patients for whether they are at high risk of being diagnosed with TBI. As training data, we are using clinical notes to retrospectively extract patient characteristics. A meta-concept has to be defined in terms of a yes/no question of the form "Does the note mention this patient having...?". Pick meta-concepts that are as specific as possible, without being too broad.

Our team was previously considering the following set of concepts for the CBM:
<question_list>
Does the note mention the patient experiencing a loss of consciousness?
```

```
Does the note mention the patient having a Glasgow Coma Scale score of 15?
Does the note mention the patient experiencing a headache?
Does the note mention the patient having normal cognition?
Does the note mention the patient being seizure-free?
Does the note mention the patient having a seizure?
Does the note mention the patient having any neurological symptoms?
Does the note mention the patient experiencing a cranial impact?
Does the note mention the patient having a fall incident?
Does the note mention the patient having a history of head trauma?
Does the note mention the patient having tachycardia?
Does the note mention the patient having no vomiting?
</question_list>

Clinical experts gave the following feedback to our concepts:
<feedback>
Rather than using concepts that depend on the status of the patient having neurological assessments, propose concepts
↪  that are characteristics of the patient themself.

Some concepts like Glasgow Coma Scale score only make sense if a patient is at least 2 years old, or have different
↪  interpretations if the patient is above or below 2 years old. For such questions, write them as a conjunction,
↪  e.g. "Does the note mention the patient having a Glasgow Coma Scale score of 15 and being at least 2 years old?"

Consider adding questions related to whether a patient has a hematoma and its location on the head.

Concepts they particularly liked or suggested including:
Does the note mention the patient experiencing loss of consciousness?
Does the note mention the patient having a cognitive delay?
Does the note mention the patient having a history of head trauma?
Does the note mention the patient having vomiting or nausea?
</feedback>

We also conducted a data-driven analysis of the top words most associated with TBI as estimated using a logistic
↪  regression model with tf-idf features.
Words with top coefficients:

{top_features_df}

YOUR TASK:
Propose {max_meta_concepts} candidate meta-concepts for a CBM. Do this by revising the original list of concepts,
↪  incorporating expert feedback, and incorporating the results from the data-driven analyses. You can merge, revise,
↪  or add new questions. Systematically review the feedback and the tf-idf top words list. Prioritize concepts that
↪  appear in the tf-idf results and align with the feedback guidance.

Suggestions for generating candidate meta-concepts:
Do not propose meta-concepts that are simply a union of two different concepts (e.g. "Does the note mention this
↪  patient having an infection or being elderly?" is not allowed), questions with answers that are almost always a
↪  yes (e.g. the answer to "Does the note mention this patient being hospitalized?" is almost always yes), or
↪  questions where the yes/no options are not clearly defined (e.g. "Does the note mention this patient experiencing
↪  difficulty?" is not clearly defined because difficulty may mean financial difficulty, physical difficulties, etc).
Rather than using concepts that depend on the status of the patient having neurological assessments, propose concepts
↪  that are characteristics of the patient themself.
Some concepts like Glasgow Coma Scale score only make sense if a patient is at least 2 years old, or have different
↪  interpretations if the patient is above or below an 2 years old. For such questions, write them as a conjunction,
↪  e.g. "Does the note mention the patient having a Glasgow Coma Scale score of 15 and being at least 2 years old?"
Concepts that clinical experts have suggested include those relating to loss of consciousness, cognitive delays,
↪  history of traumatic brain injury, vomiting, headaches, hematoma characteristics

Propose at least {max_meta_concepts} candidates in the following JSON format:
{
  "reasoning": "Step-by-step explanation of how you systematically worked through the model concepts (from most to
  ↪  least predictive).",
  "concepts": [
    {
      "concept": "<QUESTION 1>",
      "words": ["<SYNONYM/ANTONYM 1>", "<SYNONYM/ANTONYM 2>", "<SYNONYM/ANTONYM 3>"]
    },
    {
      "concept": "<QUESTION 2>",
      "words": ["<SYNONYM/ANTONYM 1>", "<SYNONYM/ANTONYM 2>", "<SYNONYM/ANTONYM 3>"]
    },
    ...
    {
      "concept": "<QUESTION {max_meta_concepts}>",
```

```
      "words": ["<SYNONYM/ANTONYM 1>", "<SYNONYM/ANTONYM 2>", "<SYNONYM/ANTONYM 3>"]
    }
  ]
}

Example answer:
{
  "reasoning": "Step-by-step reasoning through the residual model concepts and feedback",
  "concepts": [
    {
      "concept": "Does the note mention the patient experiencing head trauma?",
      "words": ["head injury", "skull trauma", "cranial damage"]
    },
    {
      "concept": "Does the note mention the patient experiencing loss of consciousness?",
      "words": ["LOC", "fainting", "unresponsive", "syncope"]
    },
    {
      "concept": "Does the note mention the patient having a cognitive delay?",
      "words": ["cognitive impairment", "intellectual disability", "developmental delay"]
    },
    {
      "concept": "Does the note mention the patient having altered mental status?",
      "words": ["mental status changes", "altered level of consciousness", "confusion", "disorientation", ]
    }
    ]
}


Final answer:
```

### E.2.3 ConceptReplacementProposalPrompt

```
The goal is to come up with a concept bottleneck model (CBM) that extracts {num_concepts} meta-concepts from clinical
↪  notes from the electronic health record of pediatric patients admitted to the emergency room to predict risk of
↪  having a traumatic brain injury (TBI).  A meta-concept is a binary feature extractor defined by a yes/no question
↪  of the form "Does the note mention this patient having...?". We have {num_concepts_fixed} meta-concepts so far:
{meta_concepts}

To come up with the {num_concepts}th meta-concept, I have done the following: I first fit a CBM on the
↪  {num_concepts_fixed} existing meta-concepts. Then to figure out how to improve this {num_concepts_fixed}-concept
↪  CBM, I first extracted a list of concepts that are present in each note, and then fit a linear regression model on
↪  the extracted concepts to predict the residuals of the {num_concepts_fixed}-concept CBM. These are the top
↪  extracted concepts in the resulting residual model, in descending order of importance:

{top_features_df}

To interpret this residual model, a general rule of thumb is that an extracted concept with a large positive
↪  coefficient means that a high risk of TBI  is positively associated with the concept being mentioned in the note,
↪  and an extracted concept with a negative coefficient means that a higher risk of TBI is negatively associated with
↪  the concept being mentioned in the note.

Given the residual model above, create cohesive candidates for the {num_concepts}th meta-concept. Be systematic and
↪  consider all the listed concepts in the residual model. Start from the most to the least predictive concept. For
↪  each concept, check if it matches an existing meta-concept or create a new candidate meta-concept. Work down the
↪  list, iterating through each concept. Clearly state each candidate meta-concept as a yes/no question.

Suggestions for generating candidate meta-concepts:
* Do not propose meta-concepts that are simply a union of two different concepts (e.g. "Does the note mention this
↪  patient having an infection or being elderly?" is not allowed), questions with answers that are almost always a
↪  yes (e.g. the answer to "Does the note mention this patient being hospitalized?" is almost always yes), or
↪  questions where the yes/no options are not clearly defined (e.g. "Does the note mention this patient experiencing
↪  difficulty?" is not clearly defined because difficulty may mean financial difficulty, physical difficulties, etc).
* Do not propose meta-concepts where you would expect over 95% agreement or disagreement with the {num_concepts_fixed}
↪  existing meta-concepts (e.g. "Does the note mention the patient having homelessness?" overlaps too much with "Does
↪  the note mention the patient having housing insecurity?").
* Rather than using concepts that depend on the status of the patient having neurological assessments, propose
↪  concepts that are characteristics of the patient themself.
* Some concepts like Glasgow Coma Scale score only make sense if a patient is at least 2 years old, or have different
↪  interpretations if the patient is above or below 2 years old. For such questions, write them as a conjunction,
↪  e.g. "Does the note mention the patient having a Glasgow Coma Scale score of 15 and being at least 2 years old?"
```

```
* Concepts that clinical experts have suggested include those relating to loss of consciousness, cognitive delays, history of traumatic brain injury, vomiting, headaches, hematoma characteristics
* Do NOT propose concepts like "Does the note mention the patient having a low risk of TBI?" or "Does the note mention the patient having a high risk of TBI?" Our goal is to learn specific risk factors or protective factors of TBI.

Finally, summarize all the generated candidates for the {num_concepts}-th meta-concept in a JSON. Merge any candidate meta-concepts that are essentially the same (where you would expect over 95% agreement) or essentially opposites (you would expect over 95% disagreement). In the JSON, include a list of comma-separated list of phrases that mean the same or opposite of each candidate meta-concept. Propose at least ten candidates. The final JSON should have the following format:

Answer with a list of {num_meta_concepts} JSONs, one per line:
{
  "reasoning": "Step-by-step explanation of how you systematically worked through the residual model concepts (from most to least predictive).",
  "concepts": [
    {
      "concept": "<QUESTION 1>",
      "words": ["<SYNONYM/ANTONYM 1>", "<SYNONYM/ANTONYM 2>", "<SYNONYM/ANTONYM 3>"]
    },
    {
      "concept": "<QUESTION 2>",
      "words": ["<SYNONYM/ANTONYM 1>", "<SYNONYM/ANTONYM 2>", "<SYNONYM/ANTONYM 3>"]
    },
    ...
    {
      "concept": "<QUESTION {num_meta_concepts}>",
      "words": ["<SYNONYM/ANTONYM 1>", "<SYNONYM/ANTONYM 2>", "<SYNONYM/ANTONYM 3>"]
    }
  ]
}


Example answer:
{
  "reasoning": "Step-by-step reasoning through the residual model concepts",
  "concepts": [
    {
      "concept": "Does the note mention the patient experiencing head trauma?",
      "words": ["head injury", "skull trauma", "cranial damage"]
    },
    {
      "concept": "Does the note mention the patient experiencing loss of consciousness?",
      "words": ["LOC", "fainting", "unresponsive", "syncope"]
    },
    {
      "concept": "Does the note mention the patient having a cognitive delay?",
      "words": ["cognitive impairment", "intellectual disability", "developmental delay"]
    },
    {
      "concept": "Does the note mention the patient having altered mental status?",
      "words": ["mental status changes", "altered level of consciousness", "confusion", "disorientation", ]
    }
    ]
}
```

### E.2.4 ConceptAnnotationPrompt

```
You will be given clinical notes. I will give you a series of questions. Your task is answer each question with a probability from 0 to 1. Summarize the response with a JSON that includes your answer to all of the questions.

Questions:
{prompt_questions}

Clinical notes:
{sentence}

Example answer: {
    "0": 0,
    "1": 1,
    "2": 0.5
```

```
}
Answer all the questions and do not answer with anything else besides valid JSON. Do not add comments to the JSON.
```

## E.3 Round 3

### E.3.1 KeyphrasePrompt

```
You are extracting clinical descriptors of a pediatric patient from emergency room medical notes that may influence their risk of being diagnosed with traumatic brain injury (TBI).  Focus entirely on factors explicitly mentioned in the notes. Do not infer or add details unless explicitly stated.

Extract factors that may:
- Increase TBI risk, be specific and detailed (e.g., substance use, prior head injuries, loss of consciousness, cognitive delays, history of traumatic brain injury, vomiting, headaches, hematoma, location of hematoma, GCS<15)
- Decrease TBI risk, be specific and detailed (e.g., protective equipment use, medication compliance, GCS=15)
- Indicate TBI severity or complications, be specific and detailed (e.g., seizures, cognitive changes, somnolence)

For each factor, provide:
1. The primary descriptor (exact or paraphrased from the note)
2. Synonyms or alternative phrasings
3. Broader generalizations that capture similar concepts

Requirements:
- Each descriptor should be $\leq$3 words
- The descriptor should state distinguishing features. Focus on clinically relevant factors.
- List specific terms, synonyms, and increasingly general concepts. However, do NOT state overly general concepts like "protective factor", "risk factor", "low risk", or "high risk" because we are using these terms to brainstorm/learn specific factors that clinicians can use to predict TBI risk.
- Do not extract concepts or list generalizations of concepts that simply describe patients having a neurological assessment. Instead propose concepts that are the results from such an assessment and/or characteristics or symptoms of the patient themself.
- Separate items with commas

Note(s):
"{note}"

Do not infer or fabricate details. Base all responses strictly on the medical note text.

Output Format:
{
    "reasoning": "Describe any reasoning here",
    "keyphrases": [
        "Primary descriptor, Synonym1, Synonym2, Broader category",
        "Primary descriptor, Alternative phrasing, More general concept",
        ...
    ]
}

Examples:
{
    "reasoning": "Describe any reasoning here",
    "keyphrases": [
        "Crowded residence, Overcrowded living",
        "Cervical strain, Neck pain, Muscle spasm",
        "Physical assault, Blunt trauma",
        "Vomiting, emesis",
        "GCS=15, normal GCS",
        ...
    ]
}
```

### E.3.2 ConceptInitializationProposalPrompt

```
We are fitting a concept bottleneck model to predict traumatic brain injury (TBI) for pediatric patients admitted to emergency room. The goal is to come up with a few meta-concepts that clinicians can use to easily risk-stratify patients for whether they are at high risk of being diagnosed with TBI. As training data, we are using clinical notes to retrospectively extract patient characteristics. A meta-concept has to be defined in terms of a yes/no question of the form "Does the note mention this patient having...?". Pick meta-concepts that are as specific as possible, without being too broad.

Our team was previously considering the following set of concepts for the CBM:
```

```
<question_list>
Does the note mention the patient experiencing a loss of consciousness?
Does the note mention the patient having a Glasgow Coma Scale score of 15 and being at least 2 years old?
Does the note mention the patient having altered mental status?
Does the note mention the patient experiencing a headache?
Does the note mention the patient having normal cognition?
Does the note mention the patient having intact cranial nerves?
Does the note mention the patient experiencing convulsions?
Does the note mention the patient experiencing drowsiness?
Does the note mention the patient experiencing a behavioral change?
Does the note mention the patient being seizure-free?
Does the note mention the patient having a seizure?
Does the note mention the patient having any neurological symptoms?
Does the note mention the patient experiencing a cranial impact?
Does the note mention the patient having a fall incident?
Does the note mention the patient having a history of head trauma?
Does the note mention the patient having tachycardia?
Does the note mention the patient having no vomiting?
</question_list>

Clinical experts gave the following feedback to our concepts:
<feedback>
Rather than using concepts that depend on the status of the patient having neurological assessments, propose concepts
↪  that are characteristics of the patient themself.

Some concepts like Glasgow Coma Scale score only make sense if a patient is at least 2 years old, or have different
↪  interpretations if the patient is above or below 2 years old. For such questions, write them as a conjunction,
↪  e.g. "Does the note mention the patient having a Glasgow Coma Scale score of 15 and being at least 2 years old?"

Consider adding questions related to whether a patient has a hematoma and its location on the head.

Concepts they particularly liked or suggested including:
Does the note mention the patient experiencing loss of consciousness?
Does the note mention the patient having a cognitive delay?
Does the note mention the patient having a history of head trauma?
Does the note mention the patient having vomiting or nausea?
</feedback>

We also conducted a data-driven analysis of the top words most associated with TBI as estimated using a logistic
↪  regression model with tf-idf features.
Words with top coefficients:

{top_features_df}

YOUR TASK:
Propose {max_meta_concepts} candidate meta-concepts for a CBM. Do this by revising the original list of concepts,
↪  incorporating expert feedback, and incorporating the results from the data-driven analyses. You can merge, revise,
↪  or add new questions. Systematically review the feedback and the tf-idf top words list. Prioritize concepts that
↪  appear in the tf-idf results and align with the feedback guidance.

Suggestions for generating candidate meta-concepts:
* Do not propose meta-concepts that are simply a union of two different concepts (e.g. "Does the note mention this
↪  patient having an infection or being elderly?" is not allowed), questions with answers that are almost always a
↪  yes (e.g. the answer to "Does the note mention this patient being hospitalized?" is almost always yes), or
↪  questions where the yes/no options are not clearly defined (e.g. "Does the note mention this patient experiencing
↪  difficulty?" is not clearly defined because difficulty may mean financial difficulty, physical difficulties, etc).
* Rather than using concepts that depend on the status of the patient having neurological assessments, propose
↪  concepts that are characteristics of the patient themself or the results of such an assessment.
* Some concepts like Glasgow Coma Scale score only make sense if a patient is at least 2 years old, or have different
↪  interpretations if the patient is above or below an 2 years old. For such questions, write them as a conjunction,
↪  e.g. "Does the note mention the patient having a Glasgow Coma Scale score of 15 and being at least 2 years old?"
* Concepts that clinical experts have suggested include those relating to loss of consciousness, cognitive delays,
↪  history of traumatic brain injury, vomiting, headaches, hematoma characteristics
* Do NOT propose concepts like "Does the note mention the patient having a low risk of TBI?" or "Does the note mention
↪  the patient having a high risk of TBI?" Our goal is to learn specific risk factors or protective factors of TBI.

Propose at least {max_meta_concepts} candidates in the following JSON format:
{
  "reasoning": "Step-by-step explanation of how you systematically worked through the model concepts (from most to
  ↪  least predictive).",
  "concepts": [
    {
      "concept": "<QUESTION 1>",
```

```
      "words": ["<SYNONYM/ANTONYM 1>", "<SYNONYM/ANTONYM 2>", "<SYNONYM/ANTONYM 3>"]
    },
    {
      "concept": "<QUESTION 2>",
      "words": ["<SYNONYM/ANTONYM 1>", "<SYNONYM/ANTONYM 2>", "<SYNONYM/ANTONYM 3>"]
    },
    ...
    {
      "concept": "<QUESTION {max_meta_concepts}>",
      "words": ["<SYNONYM/ANTONYM 1>", "<SYNONYM/ANTONYM 2>", "<SYNONYM/ANTONYM 3>"]
    }
  ]
}

Example answer:
{
  "reasoning": "Step-by-step reasoning through the residual model concepts and feedback",
  "concepts": [
    {
      "concept": "Does the note mention the patient experiencing head trauma?",
      "words": ["head injury", "skull trauma", "cranial damage"]
    },
    {
      "concept": "Does the note mention the patient experiencing loss of consciousness?",
      "words": ["LOC", "fainting", "unresponsive", "syncope"]
    },
    {
      "concept": "Does the note mention the patient having a cognitive delay?",
      "words": ["cognitive impairment", "intellectual disability", "developmental delay"]
    },
    {
      "concept": "Does the note mention the patient having altered mental status?",
      "words": ["mental status changes", "altered level of consciousness", "confusion", "disorientation", ]
    }
    ]
}


Final answer:
```

### E.3.3 ConceptReplacementProposalPrompt

```
The goal is to come up with a concept bottleneck model (CBM) that extracts {num_concepts} meta-concepts from clinical
↪  notes from the electronic health record of pediatric patients admitted to the emergency room to predict risk of
↪  having a traumatic brain injury (TBI).  A meta-concept is a binary feature extractor defined by a yes/no question
↪  of the form "Does the note mention this patient having...?". We have {num_concepts_fixed} meta-concepts so far:
{meta_concepts}

To come up with the {num_concepts}th meta-concept, I have done the following: I first fit a CBM on the
↪  {num_concepts_fixed} existing meta-concepts. Then to figure out how to improve this {num_concepts_fixed}-concept
↪  CBM, I first extracted a list of concepts that are present in each note, and then fit a linear regression model on
↪  the extracted concepts to predict the residuals of the {num_concepts_fixed}-concept CBM. These are the top
↪  extracted concepts in the resulting residual model, in descending order of importance:

{top_features_df}

To interpret this residual model, a general rule of thumb is that an extracted concept with a large positive
↪  coefficient means that a high risk of TBI  is positively associated with the concept being mentioned in the note,
↪  and an extracted concept with a negative coefficient means that a higher risk of TBI is negatively associated with
↪  the concept being mentioned in the note.

Given the residual model above, create cohesive candidates for the {num_concepts}th meta-concept. Be systematic and
↪  consider all the listed concepts in the residual model. Start from the most to the least predictive concept. For
↪  each concept, check if it matches an existing meta-concept or create a new candidate meta-concept. Work down the
↪  list, iterating through each concept. Clearly state each candidate meta-concept as a yes/no question.

Suggestions for generating candidate meta-concepts:
* Do not propose meta-concepts that are simply a union of two different concepts (e.g. "Does the note mention this
↪  patient having an infection or being elderly?" is not allowed), questions with answers that are almost always a
↪  yes (e.g. the answer to "Does the note mention this patient being hospitalized?" is almost always yes), or
↪  questions where the yes/no options are not clearly defined (e.g. "Does the note mention this patient experiencing
↪  difficulty?" is not clearly defined because difficulty may mean financial difficulty, physical difficulties, etc).
```

```
* Do not propose meta-concepts where you would expect over 95% agreement or disagreement with the {num_concepts_fixed} existing meta-concepts (e.g. "Does the note mention the patient having homelessness?" overlaps too much with "Does the note mention the patient having housing insecurity?").
* Rather than using concepts that depend on the status of the patient having neurological assessments, propose concepts that are characteristics of the patient themself.
* Some concepts like Glasgow Coma Scale score only make sense if a patient is at least 2 years old, or have different interpretations if the patient is above or below 2 years old. For such questions, write them as a conjunction, e.g. "Does the note mention the patient having a Glasgow Coma Scale score of 15 and being at least 2 years old?"
* Concepts that clinical experts have suggested include those relating to loss of consciousness, cognitive delays, history of traumatic brain injury, vomiting, headaches, hematoma characteristics
* Do NOT propose concepts like "Does the note mention the patient having a low risk of TBI?" or "Does the note mention the patient having a high risk of TBI?" or "Does the note mention the patient having symptoms of TBI?". The instructions are to learn specific risk factors or protective factors of TBI.

Finally, summarize all the generated candidates for the {num_concepts}-th meta-concept in a JSON. Merge any candidate meta-concepts that are essentially the same (where you would expect over 95% agreement) or essentially opposites (you would expect over 95% disagreement). In the JSON, include a list of comma-separated list of phrases that mean the same or opposite of each candidate meta-concept. Propose at least ten candidates. The final JSON should have the following format:

Answer with a list of {num_meta_concepts} JSONs, one per line:
{
  "reasoning": "Step-by-step explanation of how you systematically worked through the residual model concepts (from most to least predictive).",
  "concepts": [
    {
      "concept": "<QUESTION 1>",
      "words": ["<SYNONYM/ANTONYM 1>", "<SYNONYM/ANTONYM 2>", "<SYNONYM/ANTONYM 3>"],
      "is_risk_factor": <bool: True if a yes answer to this concept question is a risk factor, False if a yes answer to this concept question is a protective factor>
    },
    {
      "concept": "<QUESTION 2>",
      "words": ["<SYNONYM/ANTONYM 1>", "<SYNONYM/ANTONYM 2>", "<SYNONYM/ANTONYM 3>"],
      "is_risk_factor": <bool: True if a yes answer to this concept question is a risk factor, False if a yes answer to this concept question is a protective factor>
    },
    ...
    {
      "concept": "<QUESTION {num_meta_concepts}>",
      "words": ["<SYNONYM/ANTONYM 1>", "<SYNONYM/ANTONYM 2>", "<SYNONYM/ANTONYM 3>"],
      "is_risk_factor": <bool: True if a yes answer to this concept question is a risk factor, False if a yes answer to this concept question is a protective factor>
    }
  ]
}


Example answer:
{
  "reasoning": "Step-by-step reasoning through the residual model concepts",
  "concepts": [
    {
      "concept": "Does the note mention the patient experiencing head trauma?",
      "words": ["head injury", "skull trauma", "cranial damage"],
      "is_risk_factor": True
    },
    {
      "concept": "Does the note mention the patient experiencing loss of consciousness?",
      "words": ["LOC", "fainting", "unresponsive", "syncope"],
      "is_risk_factor": True
    },
    {
      "concept": "Does the note mention the patient having a cognitive delay?",
      "words": ["cognitive impairment", "intellectual disability", "developmental delay"],
      "is_risk_factor": True
    },
    {
      "concept": "Does the note mention the patient having altered mental status?",
      "words": ["mental status changes", "altered level of consciousness", "confusion", "disorientation"],
      "is_risk_factor": True
    },
    {
```

```
      "concept": "Does the note mention the patient is alert and responsive?",
      "words": ["normal cognitive status", "alert", "normal cognition", "normal response time"],
      "is_risk_factor": False
    }
    ]
}
```

### E.3.4 ConceptAnnotationPrompt

```
You will be given clinical notes. I will give you a series of questions. Your task is answer each question with a
↪  probability from 0 to 1. Summarize the response with a JSON that includes your answer to all of the questions.

Questions:
{prompt_questions}

Clinical notes:
{sentence}

Example answer: {
    "0": 0,
    "1": 1,
    "2": 0.5
}
Answer all the questions and do not answer with anything else besides valid JSON. Do not add comments to the JSON.
```

# F Prompts: AKI

## F.1 Round 1

### F.1.1 KeyphrasePrompt

```
You are extracting clinical factors from pre operative clinical notes that may influence a patient's risk of acute
↪  kidney injury when undergoing anestheia during surgery. Focus entirely on factors explicitly mentioned in the
↪  notes. Do not infer or add details unless explicitly stated.

Extract factors that may:
- Increase AKI risk, be specific and detailed (e.g., diabetes, obesity)
- Decrease AKI risk, be specific and detailed (e.g., frequent urine output, normal creatinine)
- Indicate AKI severity or complications, be specific and detailed (e.g., doubling creatinine, swelling)

For each factor, provide:
1. The primary descriptor (exact or paraphrased from the note)
2. Synonyms or alternative phrasings
3. Broader generalizations that capture similar concepts

Requirements:
- Each descriptor should be $\leq$3 words
- Focus on clinically relevant factors
- List specific terms, synonyms, and increasingly general concepts. However, do not state overly general concepts like
↪  "protective factor", "risk factor", "low risk", or "high risk" because we are using these terms to
↪  brainstorm/learn specific factors that clinicians can use to predict AKI risk.
- Separate items with commas

Note(s):
"{note}"

Do not infer or fabricate details. Base all responses strictly on the medical note text.

{
    "reasoning": "Describe any reasoning here",
    "keyphrases": [
        "Primary descriptor, Synonym1, Synonym2, Broader category",
        "Primary descriptor, Alternative phrasing, More general concept",
        ...
    ]
}

Examples:
{
    "reasoning": "Describe any reasoning here",
```

```
    "keyphrases": [
        "Crowded residence, Overcrowded living, Constrained environment, Environmental risk",
        "Cervical strain, Neck pain, Muscle spasm",
        "Physical assault, Blunt trauma, Injury infliction",
        ...
    ]
}
```

### F.1.2 ConceptInitializationProposalPrompt

```
We are fitting a concept bottleneck model to predict acute kidney injury (AKI) risk for patients undergoing anestheia
↪  during surgery. The goal is to come up with a few meta-concepts that clinicians can use to easily risk-stratify
↪  patients for whether they are at high risk of developing with AKI within 7 days after surgery. As training data,
↪  we are using preoperative anesthesia notes to retrospectively extract patient characteristics.

The top words most associated with AKI were estimated using a simple logistic regression model with tf-idf features.
↪  Based on the top words, come up with {max_meta_concepts} meta-concepts. A meta-concept has to be defined in terms
↪  of a yes/no question starting with the phrase "DOes the note mention this patient...", e.g. "Does the note mention
↪  this patient experiencing stomach pain?". Pick meta-concepts that are as specific as possible, without being too
↪  broad.

Suggestions for generating candidate meta-concepts: Do not propose meta-concepts that are simply a union of two
↪  different concepts (e.g. "Does the note mention this patient having an infection or being elderly?" is not
↪  allowed), questions with answers that are almost always a yes (e.g. the answer to "Does the note mention this
↪  patient being hospitalized?" is almost always yes), or questions where the yes/no options are not clearly defined
↪  (e.g. "Does the note mention this patient experiencing difficulty?" is not clearly defined because difficulty may
↪  mean financial difficulty, physical difficulties, etc).

Words with top coefficients:

{top_features_df}

Propose at least {max_meta_concepts} candidates in the following JSON format:
{
  "reasoning": "Step-by-step explanation of how you systematically worked through the model concepts (from most to
  ↪  least predictive).",
  "concepts": [
    {
      "concept": "<QUESTION 1>",
      "words": ["<SYNONYM/ANTONYM 1>", "<SYNONYM/ANTONYM 2>", "<SYNONYM/ANTONYM 3>"]
    },
    {
      "concept": "<QUESTION 2>",
      "words": ["<SYNONYM/ANTONYM 1>", "<SYNONYM/ANTONYM 2>", "<SYNONYM/ANTONYM 3>"]
    },
    ...
    {
      "concept": "<QUESTION {max_meta_concepts}>",
      "words": ["<SYNONYM/ANTONYM 1>", "<SYNONYM/ANTONYM 2>", "<SYNONYM/ANTONYM 3>"]
    }
  ]
}

Example answer:
{
  "reasoning": "Step-by-step reasoning through the model concepts",
  "concepts": [
    {
      "concept": "Does the note mention the patient having a history of pneumonia?",
      "words": ["pneumonia", "lung infection"]
    },
    {
      "concept": \Does the note mention the patient being uninsured?"
      "words": ["lack of insurance", uninsured"]
    },
    ...
    ]
}
```

### F.1.3 ConceptReplacementProposalPrompt

```
The goal is to come up with a concept bottleneck model (CBM) that extracts {num_concepts} meta-concepts from pre operative notes to predict acute kidney injury (AKI) for patients undergoing anestheia during surgery. A meta-concept is a binary feature extractor defined by a yes/no question starting with the phrase "Does the note mention this patient...". We have {num_concepts_fixed} meta-concepts so far:
{meta_concepts}

To come up with the {num_concepts}th meta-concept, I have done the following: I first fit a CBM on the {num_concepts_fixed} existing meta-concepts. Then to figure out how to improve this {num_concepts_fixed}-concept CBM, I first extracted a list of concepts that are present in each note, and then fit a linear regression model on the extracted concepts to predict the residuals of the {num_concepts_fixed}-concept CBM. These are the top extracted concepts in the resulting residual model, in descending order of importance:

{top_features_df}

To interpret this residual model, a general rule of thumb is that an extracted concept with a large positive coefficient means that a high risk of AKI is positively associated with the concept being mentioned in the note, and an extracted concept with a negative coefficient means that a higher risk of AKI is negatively associated with the concept being mentioned in the note.

Given the residual model, create cohesive candidates for the {num_concepts}th meta-concept. Be systematic and consider all the listed concepts in the residual model. Start from the most to the least predictive concept. For each concept, check if it matches an existing meta-concept or create a new candidate meta-concept. Work down the list, iterating through each concept. Clearly state each candidate meta-concept as a yes/no question.

Suggestions for generating candidate meta-concepts: Do not propose meta-concepts that are simply a union of two different concepts (e.g. "Does the note mention this patient having an infection or being elderly?" is not allowed), questions with answers that are almost always a yes (e.g. the answer to "Does the note mention this patient being hospitalized?" is almost always yes), or questions where the yes/no options are not clearly defined (e.g. "Does the note mention this patient experiencing difficulty?" is not clearly defined because difficulty may mean financial difficulty, physical difficulties, etc). Do not propose meta-concepts where you would expect over 95% agreement or disagreement with the {num_concepts_fixed} existing meta-concepts (e.g. "Does the note mention the patient having homelessness?" overlaps too much with "Does the note mention the patient having housing insecurity?").

Finally, summarize all the generated candidates for the {num_concepts}-th meta-concept in a JSON. Merge any candidate meta-concepts that are essentially the same (where you would expect over 95% agreement) or essentially opposites (you would expect over 95% disagreement). In the JSON, include a list of comma-separated list of phrases that mean the same or opposite of each candidate meta-concept. Propose at least ten candidates. The final JSON should have the following format:

Answer with a list of {num_concepts} JSONs, one per line:
{
  "reasoning": "Step-by-step explanation of how you systematically worked through the residual model concepts (from most to least predictive).",
  "concepts": [
    {
      "concept": "<QUESTION 1>",
      "words": ["<SYNONYM/ANTONYM 1>", "<SYNONYM/ANTONYM 2>", "<SYNONYM/ANTONYM 3>"]
    },
    {
      "concept": "<QUESTION 2>",
      "words": ["<SYNONYM/ANTONYM 1>", "<SYNONYM/ANTONYM 2>", "<SYNONYM/ANTONYM 3>"]
    },
    ...
    {
      "concept": "<QUESTION {num_concepts}>",
      "words": ["<SYNONYM/ANTONYM 1>", "<SYNONYM/ANTONYM 2>", "<SYNONYM/ANTONYM 3>"]
    }
  ]
}

Example answer:
{
  "reasoning": "Step-by-step reasoning through the residual model concepts",
  "concepts": [
    {
      "concept": "Does the note mention the patient having a history of pneumonia?",
      "words": ["penumonia", "lung infection"]
    },
    {
      "concept": "Does the note mention the patient being uninsured?",
```

```
      "words": ["no insurance", "uninsured"]
    },
    ...
    ]
}
```

### F.1.4 ConceptAnnotationPrompt

```
You will be given clinical notes. I will give you a series of questions. Your task is answer each question with a
↪  probability from 0 to 1. Summarize the response with a JSON that includes your answer to all of the questions.
↪  Questions:
{prompt_questions}

clinical notes:
{sentence}

Example answer: {
    "0": 0,
    "1": 1,
    "2": 0.5
}
Answer all the questions and do not answer with anything else besides valid JSON. Do not add comments to the JSON.
```

## F.2 Round 2

### F.2.1 KeyphrasePrompt

```
You are extracting characteristics and clinical factors of a patient from pre-operative clinical notes that may
↪  influence a patient's risk of acute kidney injury (AKI) when undergoing anesthesia during surgery. Focus entirely
↪  on factors explicitly mentioned in the notes.

(A) Extract patient factors that may:
- Increase AKI risk, be specific and detailed (e.g., male, heart failure, diabetes, obesity, active infection,
↪  coronary disease, bleeding disorder, multiple medications, supplemental oxygen, intubated, low METS, limited
↪  exercise tolerance, unable to climb stairs)
- Decrease AKI risk, be specific and detailed (e.g., normal lung function, frequent urine output, normal creatinine,
↪  high METS, exercises regularly, came from home, outpatient)
- Indicate AKI severity or complications, be specific and detailed (e.g., fluid accumulation, doubling creatinine,
↪  swelling, acid reflux)

(B) Extract surgery factors that may:
- Increase AKI risk (e.g., high risk of bleeding, extended operative time, blood transfusion expected, anticipated
↪  blood loss, abdominal surgery, high risk surgery)
- Decrease AKI risk (e.g., short surgery, regional anesthesia, minimally invasive surgery, come-on-go surgery)

For each factor, provide:
1. The primary descriptor (exact or paraphrased from the note)
2. Synonyms or alternative phrasings
3. Broader generalizations that capture similar concepts

Requirements:
- Each descriptor should be $\leq$3 words
- Focus on clinically relevant factors
- Include both specific terms and generalizable concepts
- Separate items with commas
- Avoid overly general keyphrases like \gastrointestinal discomfort." Do not state overly general concepts like
↪  "protective factor", "risk factor", "low risk", or "high risk" because we are using these terms to
↪  brainstorm/learn specific factors that clinicians can use to predict AKI risk.

Note(s):
"{note}"

Do not infer or fabricate details. Base all responses strictly on the medical note text.

Output Format:
{
\reasoning": "Step-by-step reasoning through the keyphrase model concepts"
 \keyphrases": [ "Primary descriptor", \Alternative phrasing", \General concept"]
}

...
```

```
Examples:
{
\reasoning": "Your step by step reasoning"
 \keyphrases": [
        "Septic shock, sepsis, systematic inflammation",
        "Cervical strain, Neck pain, Muscle spasm",
        "Low METS, low exercise tolerance",
        ...
    ]
}
```

### F.2.2 ConceptInitializationProposalPrompt

```
We are fitting a concept bottleneck model (CBM) to predict acute kidney injury (AKI) risk for patients undergoing anesthesia during surgery. The goal is to come up with a few meta-concepts that clinicians can use to easily risk-stratify patients for whether they are at high risk of developing with AKI within 7 days after surgery. As training data, we are using preoperative anesthesia notes to retrospectively extract predictive characteristics of the patients and their planned surgeries. A meta-concept has to be defined in terms of a yes/no question of the form "Does the note mention this patient having...?" or "Does the note mention the surgery...?". Pick meta-concepts that are as specific as possible, without being too broad.

Our team was previously considering the following set of concepts for the CBM:
<question_list>
Does the note mention this patient having high serum creatinine?
Does the note mention this patient having end-stage renal disease?
Does the note mention the patient having heart failure?
Does the note mention this patient experiencing fluid retention?
Does the note mention this patient having high blood sugar?
Does the note mention this patient having a normal BMI?
Does the note mention this patient having a metabolic disorder?
</question_list>

Clinical experts gave the following feedback to our concepts:
<feedback>
* The CBM should encompass concepts from two categories: (i) characteristics of the patient and (ii) characteristics of the surgery that together influence the patient's risk of developing AKI.
* Some concepts like cardiovascular disease, coronary artery disease, and gastrointestinal discomfort are too broad. For instance, some cardiovascular diseases are life-threatening and others are almost not worth mentioning when considering the clinical outcome. Focus on concepts that distinguish between patients having low versus high risk.
* Rather than having concepts that ask if a patient has a disease diagnosis, consider concepts like \Does the note mention the patient experiencing symptoms related to ____?"
* Concepts that the expert beleives are likely relevant, but should be assessed against patterns seen in the actual data:
Does the note mention the patient having a surgery that is high risk?
Does the note mention the patient having acid reflux?
Does the note mention the patient having a normal pulmonary exam?
Does the note mention the patient having fluid accumulation?
Does the note mention the patient having heart failure?
Does the note mention the patient having less than 5.5 METS of functional capacity?
Does the note mention the patient having untreated coronary artery disease (CAD)?
Does the note mention the patient having an active infection for which they are receiving antibiotics?
Does the note mention the patient having any bleeding disorder?
Does the note mention the patient needing to be intubated or have supplemental oxygen?
Does the note mention the patient needing to take multiple medications at home?
Does the note mention the surgery being long (more than 3 hours)?
Does the note mention the surgery having a high risk of bleeding?
Does the note mention the surgery likely requiring a blood transfusion?
Does the note mention the surgery being emergent?
* Other categories of concepts clinical experts have suggested considering include:
  -   Patient characteristics: fluid accumulation, abnormal/ normal pulmonary findings, heart failure symptoms, acid reflux, bleeding disorders, abnormal laboratory values, creatinine levels, multiple medications, active infection requiring antibiotics, functional capacity (e.g., METS score, good exercise tolerance)
  - Pre-surgical status: location prior to surgery (ICU, hospital ward, or home), if the patient is intubated or needs supplemental oxygen
  - Surgical characteristics: anticipated bleeding risk, expected surgical duration, need for blood transfusion during the procedure, certain types of surgery that may be lower or higher risk for developing AKI
<feedback>

Given this feedback, we conducted an updated data analysis to find the top phrases describing either the patient and/or the planned surgery that are most associated with AKI. We did this by fitting a logistic regression model with tf-idf features.
```

```
Phrases with top coefficients:

{top_features_df}

YOUR TASK:
Propose {max_meta_concepts} candidate meta-concepts for a CBM. Do this by analyzing the results from the data-driven
↪  analyses and incorporating expert feedback, when appropriate. You can merge, revise, or add new questions.
↪  Systematically review the tf-idf top words list. Prioritize concepts that appear in the tf-idf results and align
↪  with the feedback guidance.

Do not propose meta-concepts that are simply a union of two different concepts (e.g. "Does the note mention this
↪  patient having an infection or being elderly?" is not allowed), questions with answers that are almost always a
↪  yes (e.g. the answer to "Does the note mention this patient being hospitalized?" is almost always yes), or
↪  questions where the yes/no options are not clearly defined (e.g. "Does the note mention this patient experiencing
↪  difficulty?" is not clearly defined because difficulty may mean financial difficulty, physical difficulties, etc).

Propose at least {max_meta_concepts} candidates in the following JSON format:
{
  "reasoning": "Step-by-step explanation of how you systematically worked through the residual model concepts (from
  ↪  most to least predictive) and combined them with the feedback suggestions.",
  "concepts": [
    {
      "concept": "<QUESTION 1>",
      "words": ["<SYNONYM/ANTONYM 1>", "<SYNONYM/ANTONYM 2>", "<SYNONYM/ANTONYM 3>"]
    },
    {
      "concept": "<QUESTION 2>",
      "words": ["<SYNONYM/ANTONYM 1>", "<SYNONYM/ANTONYM 2>", "<SYNONYM/ANTONYM 3>"]
    },
    ...
    {
      "concept": "<QUESTION {num_meta_concepts}>",
      "words": ["<SYNONYM/ANTONYM 1>", "<SYNONYM/ANTONYM 2>", "<SYNONYM/ANTONYM 3>"]
    }
  ]
}


Example answer:
{
  "reasoning": "Step-by-step reasoning through the residual model concepts and feedback",
  "concepts": [
    {
      "concept": "Does the note mention the patient having fluid accumulation?",
      "words": [\fluid buildup", \fluid collection", \dehydration"]
    },
    {
      "concept": \Does the note mention the patient having a normal pulmonary exam?"
      "words": [\clear lungs", \wheezing, \lungs clear"]
    },
    {
      "concept": "Does the note mention the patient having heart failure?",
      "words": [\CHF", \cardiac failure", \HF"]
    },
    {
      "concept": "Does the note mention the patient having a surgery with a high risk of bleeding?",
      "words": ["risk of bleeding", "bleeding risk"]
    }
    ...
    ]
}

Final answer:
```

### F.2.3 ConceptReplacementProposalPrompt

```
The goal is to come up with a concept bottleneck model (CBM) that extracts {num_concepts} meta-concepts from clinical
↪  notes from the electronic health record of pediatric patients admitted to the emergency room to predict acute
↪  kidney injury (AKI) for patients undergoing anesthesia during surgery. A meta-concept is a binary feature
↪  extractor defined by a yes/no question of the form "Does the note mention this patient having...?" or \Does the
↪  note mention this surgery...?". We have {num_concepts_fixed} meta-concepts so far:
{meta_concepts}
```

```
To come up with the {num_concepts}th meta-concept, I have done the following: I first fit a CBM on the {num_concepts_fixed} existing meta-concepts. Then to figure out how to improve this {num_concepts_fixed}-concept CBM, I extracted a list of concepts that are present in each note, and then fit a linear regression model on the extracted concepts to predict the residuals of the {num_concepts_fixed}-concept CBM. These are the top extracted concepts in the resulting residual model, in descending order of importance:

{top_features_df}

To interpret this residual model, a general rule of thumb is that an extracted concept with a large positive coefficient means that a high risk of AKI is positively associated with the concept being mentioned in the note, and an extracted concept with a negative coefficient means that a higher risk of AKI is negatively associated with the concept being mentioned in the note.

Given the residual model above, create cohesive candidates for the {num_concepts}th meta-concept. Be systematic and consider all the listed concepts in the residual model. Start from the most to the least predictive concept. For each concept, check if it matches an existing meta-concept or create a new candidate meta-concept. Work down the list, iterating through each concept. Clearly state each candidate meta-concept as a yes/no question.

Suggestions for generating candidate meta-concepts:
* Do not propose meta-concepts that are simply a union of two different concepts (e.g. "Does the note mention this patient having an infection or being elderly?" is not allowed), questions with answers that are almost always a yes (e.g. the answer to "Does the note mention this patient being hospitalized?" is almost always yes), or questions where the yes/no options are not clearly defined (e.g. "Does the note mention this patient experiencing difficulty?" is not clearly defined because difficulty may mean financial difficulty, physical difficulties, etc).
* Do not propose meta-concepts where you would expect over 95% agreement or disagreement with the {num_concepts_fixed} existing meta-concepts (e.g. "Does the note mention the patient having homelessness?" overlaps too much with "Does the note mention the patient having housing insecurity?").
* The CBM should encompass concepts from two categories: (i) characteristics of the patient and (ii) characteristics of the surgery that together influence the patient's risk of developing AKI.
* Some concepts like cardiovascular disease, coronary artery disease, and gastrointestinal discomfort are too broad. For instance, some cardiovascular diseases are life-threatening and others are almost not worth mentioning when considering the clinical outcome. Focus on concepts that distinguish between patients having low versus high risk.
* Rather than having concepts that ask if a patient has a disease diagnosis, consider concepts like \Does the note mention the patient experiencing symptoms related to ____?"
* Concepts that clinical experts suggested considering include:
 - Patient characteristics: fluid accumulation, abnormal/ normal pulmonary findings, heart failure symptoms, acid reflux, bleeding disorders, abnormal laboratory values, creatinine levels, multiple medications, active infection requiring antibiotics, functional capacity (e.g., METS score, good exercise tolerance), untreated coronary artery disease
 - Pre-surgical status: location prior to surgery (ICU, hospital ward, or home), if the patient is intubated or needs supplemental oxygen
 - Surgical characteristics: anticipated bleeding risk, expected surgical duration, need for blood transfusion during the procedure, certain types of surgery that may be lower or higher risk for developing AKI

Propose at least ten candidates. Answer with a list of JSONs, one per line:
Finally, summarize all the generated candidates for the {num_concepts}-th meta-concept in a JSON. Merge any candidate meta-concepts that are essentially the same (where you would expect over 95% agreement) or essentially opposites (you would expect over 95% disagreement). In the JSON, include a list of comma-separated list of phrases that mean the same or opposite of each candidate meta-concept. The final JSON should have the following format:

Answer in the following JSON format:
{
  "reasoning": "Step-by-step explanation of how you systematically worked through the residual model concepts (from most to least predictive).",
  "concepts": [
    {
      "concept": "<QUESTION 1>",
      "words": ["<SYNONYM/ANTONYM 1>", "<SYNONYM/ANTONYM 2>", "<SYNONYM/ANTONYM 3>"]
    },
    {
      "concept": "<QUESTION 2>",
      "words": ["<SYNONYM/ANTONYM 1>", "<SYNONYM/ANTONYM 2>", "<SYNONYM/ANTONYM 3>"]
    },
    ...
    {
      "concept": "<QUESTION 10>",
      "words": ["<SYNONYM/ANTONYM 1>", "<SYNONYM/ANTONYM 2>", "<SYNONYM/ANTONYM 3>"]
    }
  ]
}
```

```
Example answer:
{
  "reasoning": "Step-by-step reasoning through the residual model concepts",
   "concepts": [
    {
      "concept": "Does the note mention the patient having fluid accumulation?",
      "words": [\fluid buildup", \fluid collection", \dehydration"]
    },
    {
      "concept": \Does the note mention the patient having a normal pulmonary exam?"
      "words": [\clear lungs", \wheezing, \lungs clear"]
    },
    {
      "concept": "Does the note mention the patient having heart failure?",
      "words": [\CHF", \cardiac failure", \HF"]
    },
    ...
]
}
```

### F.2.4 ConceptAnnotationPrompt

```
You will be given clinical notes and a series of yes/no questions. For each question, answer with 1 (yes) if the
↪  information is mentioned in the note, or 0 (no) if it is not. Provide your answers in JSON format with each
↪  question mapped to its corresponding answer (0 or 1) Questions:
{prompt_questions}

clinical notes:
{sentence}

Example answer: {
    "0": 0,
    "1": 1,
    "2": 0
}
Answer all the questions and do not answer with anything else besides valid JSON. Do not add comments to the JSON.
```

## F.3 Round 3

### F.3.1 KeyphrasePrompt

```
You are extracting characteristics and clinical factors of a patient from pre-operative clinical notes that may
↪  influence a patient's risk of acute kidney injury (AKI) when undergoing anesthesia during surgery. Focus entirely
↪  on factors explicitly mentioned in the notes.

(A) Extract patient factors that may:
- Increase AKI risk, be specific and detailed (e.g., male, elderly, chronic kidney disease, renal impairment, elevated
↪  creatinine, cardiac volume overload, pulmonary edema, pitting lower extremity edema, jugular venous distension,
↪  congestive heart failure exacerbations, hepatic fluid accumulation, ascites, abdominal distension, decompensated
↪  heart failure, orthopnea, paroxysmal nocturnal dyspnea, ejection fraction < 40%, elevated BNP, active CAD,
↪  untreated CAD, angina, chest pain, poor functional capacity, <4 METs, inability to climb stairs, severe
↪  gastroesophageal reflux, active systemic infection, sepsis, bacteremia, therapeutic antibiotics, came from ICU,
↪  bleeding diathesis,  coagulopathy, hemophilia, elevated INR, low hemoglobin, anemia, intubated, home oxygen use,
↪  tracheostomy, elevated INR, low platelet count, abnormal potassium levels, uncontrolled hyperglycemia, multiple
↪  medications, recent blood transfusions, mechanical ventilation, ARDS)
- Decrease AKI risk, be specific and detailed (e.g., normal pulmonary function, lungs clear to auscultation, good
↪  exercise tolerance, >= 4 METs, ability to climb two flights of stairs, came from home, outpatient status, normal
↪  creatinine, normal liver function, hemodynamically stable, normal lung function, normal cardiac function)
- Indicate AKI severity or complications, be specific and detailed (e.g., doubling creatinine, swelling, requirement
↪  for paracentesis, febrile, requiring intensive care)

(B) Extract surgery factors that may:
- Increase AKI risk (e.g., high risk of major blood loss, abdominal surgery, liver transplantation, Whipple procedure,
↪  anticipated blood loss > 500mL, high risk of blood transfusion, major trauma surgery, transplant surgery, expected
↪  duration >= 3 hours, extended operative time, long surgery, general anesthesia, emergency surgery, intrathoracic
↪  surgery, intraperitoneal surgery, high risk of bleeding, blood transfusion expected, anticipated blood loss)
- Decrease AKI risk (e.g., short surgical duration, expected duration < 3 hours, regional anesthesia, monitored
↪  anesthesia care, minimally invasive surgery, outpatient surgery, local anesthesia)

(C) Extract medication the patient is taking which may:
```

```
- Increase AKI risk (e.g., nephrotoxic home medications,  NSAIDs, ACE inhibitors, ARBs, loop diuretics, vancomycin,
↪  gentamicin, pressors)

For each factor, provide:
1. The primary descriptor (exact or paraphrased from the note)
2. Synonyms or alternative phrasings
3. Broader generalizations that capture similar concepts

Requirements:
- Each descriptor should be $\leq$3 words
- Focus on clinically relevant factors
- Include both specific terms and generalizable concepts
- Separate items with commas
- Avoid overly general keyphrases like \gastrointestinal discomfort." Do not state overly general concepts like
↪  "protective factor", "risk factor", "low risk", or "high risk" because we are using these terms to
↪  brainstorm/learn specific factors that clinicians can use to predict AKI risk.

Note(s):
"{note}"

Do not infer or fabricate details. Base all responses strictly on the medical note text.

Output Format:
{
\reasoning": "Step-by-step reasoning through the keyphrase model concepts"
 \keyphrases": [ "Primary descriptor", \Alternative phrasing", \General concept"]
}

...

Examples:
{
\reasoning": "Your step by step reasoning"
 \keyphrases": [
        "Septic shock, sepsis, systematic inflammation",
        "Cervical strain, Neck pain, Muscle spasm",
        "Low METS, low exercise tolerance",
        ...
    ]
}
```

### F.3.2 ConceptInitializationProposalPrompt

```
We are fitting a concept bottleneck model (CBM) to predict acute kidney injury (AKI) risk for patients undergoing
↪  anesthesia during surgery. The goal is to come up with a few meta-concepts that clinicians can use to easily
↪  risk-stratify patients for whether they are at high risk of developing with AKI within 7 days after surgery. As
↪  training data, we are using preoperative anesthesia notes to retrospectively extract predictive characteristics of
↪  the patients and their planned surgeries. A meta-concept has to be defined in terms of a yes/no question of the
↪  form "Does the note mention this patient having...?", "Does the note mention this patient taking...?", or "Does
↪  the note mention the surgery...?". Pick meta-concepts that are as specific as possible, without being too broad.

Our team was previously considering the following set of concepts for the CBM:
<question_list>
Does the note mention the patient having chronic kidney disease?
Does the note mention the patient having renal impairment?
Does the note mention the patient undergoing minimally invasive surgery?
Does the note mention the patient undergoing an exploratory laparotomy?
Does the note mention the patient having sepsis?
Does the note mention the patient having an active infection requiring antibiotics?
Does the note mention the patient requiring a blood transfusion during surgery?
Does the note mention the patient having fluid retention?
Does the note mention the patient having an active lifestyle?
Does the note mention the patient having a metabolic disorder?
Does the note mention the patient having normal lung function?
Does the note mention the patient having diabetes?
Does the note mention the patient having a malignancy?
Does the note mention the patient having heart disease?
Does the note mention the surgery being high-risk?
</question_list>

Clinical experts gave the following feedback to our concepts:
<feedback>
```

```
* The CBM should encompass concepts from three categories: (i) characteristics of the patient, (ii) medications the
↪  patient is taking, and (iii) characteristics of the surgery that together influence the patient’s risk of
↪  developing AKI. Up to now, questions regarding medications have not been considered.

* Concepts must be granular and clinically precise. "High risk" or "Heart disease" or "Respiratory conditions" is too
↪  vague. You must propose concepts that include clear examples or clinical definitions to guide annotation. Concepts
↪  must be written of the format "Does the note mention the patient having ..., e.g., ...?" For instance, phrase
↪  questions like "Does the note mention the patient having good exercise tolerance, e.g. having at least 5 METS of
↪  functional capacity or ability to climb two flights of stairs?" Similarly, concepts like fluid retention should
↪  clarify if it is referring to CHF exacerbation or ascites, such as through concept questions like "Does the note
↪  mention the patient having signs of cardiac volume overload, e.g. pulmonary edema, pitting edema, or jugular
↪  venous distension?" or "Does the note mention the patient having signs of hepatic fluid accumulation, e.g. ascites
↪  or significant abdominal distension?".

* Concepts that the expert believes are likely relevant, but should be assessed against patterns seen in the actual
↪  data:
  - Does the note mention the patient having severe.e.g.,stroesophageal reflux, e.g., esophageal stricture, Barrett's
  ↪  esophagus, or a high risk of aspiration?
  - Does the note mention the patient having normal pulmonary function, e.g., mentions of lungs being clear to
  ↪  auscultation bilaterally?
  - Does the note mention the patient having signs of cardiac volume overload, e.g., pulmonary edema, pitting lower
  ↪  extremity edema, jugular venous distension (JVD), or a history of congestive heart failure exacerbations?
  - Does the note mention the patient having signs of hepatic fluid accumulation, e.g., ascites, significant abdominal
  ↪  distension, or a history of requiring paracentesis?
  - Does the note mention the patient having symptoms of decompensated Heart Failure, e.g., orthopnea, paroxysmal
  ↪  nocturnal dyspnea (PND), or a documented Ejection Fraction (EF) < 40%?
  - Does the note mention the patient having poor functional capacity, e.g., lower than 4 METs, inability to climb one
  ↪  flight of stairs, or inability to walk two blocks without shortness of breath?
  - Does the note mention the patient having active or untreated Coronary Artery Disease (CAD), e.g., current
  ↪  angina/chest pain, a recent positive stress test, or significant stenosis that has not been revascularized?
  - Does the note mention the patient having an active systemic infection, e.g., sepsis, bacteremia, fungemia, or a
  ↪  plan to administer therapeutic (non-prophylactic) antibiotics?
  - Does the note mention the patient is currently an inpatient?
  - Does the note mention the expected surgical duration is prolonged, in that it lasts longer than 3 to 4 hours?
  - Does the note mention the patient having bleeding diathesis or coagulopathy, e.g., Hemophilia, von Willebrand
  ↪  disease, liver coagulopathy (INR > 1.5), or active use of therapeutic anticoagulants (e.g.,, Warfarin,
  ↪  Apixaban)?
  - Does the note mention the patient having low hemoglobin, e.g., anemia or hemoglobin < 10 g/dL?
  - Does the note mention the patient having severe baseline respiratory compromise, e.g., the use of home oxygen,
  ↪  home CPAP/BiPAP for reasons other than sleep apnea, or a history of tracheostomy?
  - Does the note mention the surgery having a high risk of major blood loss, e.g., open abdominal aortic aneurysm
  ↪  repair, liver transplantation, Whipple procedure, or anticipated blood loss (EBL) > 500mL?
  - Does the note mention the surgery being emergent?
  - Does the note mention the surgery as having a high risk of needing a blood transfusion, e.g., major trauma surgery
  ↪  or transplant surgery?
  - Does the note mention the patient using of potentially nephrotoxic home medications, e.g., high-dose NSAIDs, ACE
  ↪  inhibitors, ARBs, chronic loop diuretics, or antibiotics like vancomycin or gentamicin?
  - Does the note mention the patient using multiple (two or more) nephrotoxic home medications, e.g., high-dose
  ↪  NSAIDs, ACE inhibitors, ARBs, chronic loop diuretics, or antibiotics like vancomycin or gentamicin?
  - Does the note mention the patient taking multiple medications at home?
* Other categories of concepts clinical experts have suggested considering include:
  - Patient characteristics: fluid accumulation, abnormal/ normal pulmonary findings, heart failure symptoms, acid
  ↪  reflux, bleeding disorders, abnormal laboratory values, creatinine levels, multiple medications, active
  ↪  infection requiring antibiotics, functional capacity (e.g., METS score, good exercise tolerance)
  - Pre-surgical status: location prior to surgery (ICU, hospital ward, or home), if the patient is intubated or needs
  ↪  supplemental oxygen
  - Surgical characteristics: anticipated bleeding risk, expected surgical duration, need for blood transfusion during
  ↪  the procedure, certain types of surgery that may be lower or higher risk for developing AKI, emergency surgery
  - Medication characteristics: multiple medications, nephrotoxic medications, antibiotics, insulin therapy
<feedback>

Given this feedback, we conducted an updated data analysis to find the top phrases describing the patient, the planned
↪  surgery, and/or the patient's medication status that are most associated with AKI. We did this by fitting a
↪  logistic regression model with tf-idf features.
Phrases with top coefficients:

{top_features_df}

YOUR TASK:
Propose {max_meta_concepts} candidate meta-concepts for a CBM. Do this by analyzing the results from the data-driven
↪  analyses and incorporating expert feedback, when appropriate. You can merge, revise, or add new questions.
↪  Systematically review the tf-idf top words list. Prioritize concepts that appear in the tf-idf results and align
↪  with the feedback guidance.
```

```
Do not propose meta-concepts that are simply a union of two different concepts (e.g. "Does the note mention this
↪  patient having an infection or being elderly?" is not allowed), questions with answers that are almost always a
↪  yes (e.g. the answer to "Does the note mention this patient being hospitalized?" is almost always yes), or
↪  questions where the yes/no options are not clearly defined (e.g. "Does the note mention this patient experiencing
↪  difficulty?" is not clearly defined because difficulty may mean financial difficulty, physical difficulties, etc).

Propose at least {max_meta_concepts} candidates in the following JSON format:
{
  "reasoning": "Step-by-step explanation of how you systematically worked through the residual model concepts (from
  ↪  most to least predictive) and combined them with the feedback suggestions.",
  "concepts": [
    {
      "concept": "<QUESTION 1>",
      "is_risk_factor": <bool: True if a yes answer to this concept question is a risk factor, False if a yes answer
      ↪  to this concept question is a protective factor>,
      "words": ["<SYNONYM/ANTONYM 1>", "<SYNONYM/ANTONYM 2>", "<SYNONYM/ANTONYM 3>"]
    },
    {
      "concept": "<QUESTION 2>",
      "is_risk_factor": <bool: True if a yes answer to this concept question is a risk factor, False if a yes answer
      ↪  to this concept question is a protective factor>,
      "words": ["<SYNONYM/ANTONYM 1>", "<SYNONYM/ANTONYM 2>", "<SYNONYM/ANTONYM 3>"]
    },
    ...
    {
      "concept": "<QUESTION {max_meta_concepts}>",
      "is_risk_factor": <bool: True if a yes answer to this concept question is a risk factor, False if a yes answer
      ↪  to this concept question is a protective factor>,
      "words": ["<SYNONYM/ANTONYM 1>", "<SYNONYM/ANTONYM 2>", "<SYNONYM/ANTONYM 3>"]
    }
  ]
}


Example answer:
{
  "reasoning": "Step-by-step reasoning through the residual model concepts and feedback",
  "concepts": [
    {
      "concept": "Does the note mention the patient having signs of hepatic fluid accumulation, e.g., ascites,
      ↪  significant abdominal distension, or need for paracentesis?",
      "is_risk_factor": True,
      "words": [\ascites", \abdominal distension"]
    },
    {
      "concept": "Does the note mention the patient having normal pulmonary function, e.g., documentation of lungs
      ↪  being clear to auscultation?",
      "is_risk_factor": False,
      "words": [\clear lungs", \wheezing, \lungs clear"]
    },
    {
      "concept": "Does the note mention the patient having poor functional capacity, e.g., having less than 4 METs and
      ↪  inability to climb stairs?",
      "is_risk_factor": True,
      "words": ["unable to climb stairs", "poor exercise tolerance", "< 4 METs"]
    },
    {
      "concept": "Does the note mention the surgery having a high risk of major blood loss, e.g., open abdominal
      ↪  aortic aneurysm repair, liver transplantation, Whipple procedure, or anticipated blood loss (EBL) > 500mL?",
      "is_risk_factor": True,
      "words": ["open abdominal aortic aneurysm repair", "anticipated blood loss (EBL) > 500mL"]
    },
    {
      "concept": "Does the note mention the patient using of potentially nephrotoxic home medications, e.g., high-dose
      ↪  NSAIDs, ACE inhibitors, ARBs, chronic loop diuretics, or antibiotics like vancomycin or gentamicin?",
      "is_risk_factor": True,
      "words": ["high-dose NSAIDs", "ACE inhibitors", "ARBs", "chronic loop diuretics", "vancomycin", "gentamicin"]
    }
    ...
    ]
}
```

```
Final answer:
```

### F.3.3 ConceptReplacementProposalPrompt

```
We are fitting a concept bottleneck model (CBM) to predict acute kidney injury (AKI) risk for patients undergoing anesthesia during surgery. The goal is to come up with a few meta-concepts that clinicians can use to easily risk-stratify patients for whether they are at high risk of developing with AKI within 7 days after surgery. As training data, we are using preoperative anesthesia notes to retrospectively extract predictive characteristics of the patients and their planned surgeries. A meta-concept has to be defined in terms of a yes/no question of the form "Does the note mention this patient having...?", "Does the note mention this patient taking...?", or "Does the note mention the surgery...?". We have {num_concepts_fixed} meta-concepts so far:
{meta_concepts}

To come up with the {num_concepts}th meta-concept, I have done the following: I first fit a CBM on the {num_concepts_fixed} existing meta-concepts. Then to figure out how to improve this {num_concepts_fixed}-concept CBM, I extracted a list of concepts that are present in each note, and then fit a linear regression model on the extracted concepts to predict the residuals of the {num_concepts_fixed}-concept CBM. These are the top extracted phrases describing the patient, the planned surgery, and/or the patient's medication status in the resulting residual model, in descending order of importance:

{top_features_df}

To interpret this residual model, a general rule of thumb is that an extracted concept with a large positive coefficient means that a high risk of AKI is positively associated with the concept being mentioned in the note, and an extracted concept with a negative coefficient means that a higher risk of AKI is negatively associated with the concept being mentioned in the note.

Given the residual model above, create cohesive candidates for the {num_concepts}th meta-concept. Be systematic and consider all the listed concepts in the residual model. Start from the most to the least predictive concept. For each concept, check if it matches an existing meta-concept or create a new candidate meta-concept. Work down the list, iterating through each concept. Clearly state each candidate meta-concept as a yes/no question.

Suggestions for generating candidate meta-concepts:
* Do not propose meta-concepts that are simply a union of two different concepts (e.g. "Does the note mention this patient having an infection or being elderly?" is not allowed), questions with answers that are almost always a yes (e.g. the answer to "Does the note mention this patient being hospitalized?" is almost always yes), or questions where the yes/no options are not clearly defined (e.g. "Does the note mention this patient experiencing difficulty?" is not clearly defined because difficulty may mean financial difficulty, physical difficulties, etc).
* Do not propose meta-concepts where you would expect over 95% agreement or disagreement with the {num_concepts_fixed} existing meta-concepts (e.g. "Does the note mention the patient having homelessness?" overlaps too much with "Does the note mention the patient having housing insecurity?").
* The CBM should consider concepts from three categories: (i) characteristics of the patient, (ii) medications the patient is taking, and (iii) characteristics of the surgery.
* Concepts must be granular and clinically precise. "High risk" or "Heart disease" is too vague. You must propose concepts that include clear examples or clinical definitions to guide annotation. Concepts are preferably written of the format "Does the note mention the patient having ..., e.g. ...?" For instance, phrase questions like "Does the note mention the patient having good exercise tolerance, e.g. having at least 5 METS of functional capacity or ability to climb two flights of stairs?" Similarly, concepts like fluid accumulation should clarify if it is referring to CHF exacerbation or ascites, such as through concept questions like "Does the note mention the patient having signs of cardiac volume overload, e.g. pulmonary edema, pitting edema, or jugular venous distension?" or "Does the note mention the patient having signs of hepatic fluid accumulation, e.g. ascites or significant abdominal distension?". Other examples from the clinician:
  - Does the note mention the patient having severe gastroesophageal reflux, e.g. esophageal stricture, Barrett's esophagus, or a high risk of aspiration?
  - Does the note mention the patient having normal pulmonary function, e.g. mentions of lungs being clear to auscultation bilaterally?
  - Does the note mention the patient having signs of cardiac volume overload, e.g. pulmonary edema, pitting lower extremity edema, jugular venous distension (JVD), or a history of congestive heart failure exacerbations?
  - Does the note mention the patient having signs of hepatic fluid accumulation, e.g. ascites, significant abdominal distension, or a history of requiring paracentesis?
  - Does the note mention the patient having symptoms of decompensated Heart Failure, e.g. orthopnea, paroxysmal nocturnal dyspnea (PND), or a documented Ejection Fraction (EF) < 40%?
  - Does the note mention the surgery having a high risk of major blood loss, e.g. open abdominal aortic aneurysm repair, liver transplantation, Whipple procedure, or anticipated blood loss (EBL) > 500mL?
  - Does the note mention the patient having poor functional capacity, e.g. lower than 4 METs, inability to climb one flight of stairs, or inability to walk two blocks without shortness of breath?
  - Does the note mention the patient having active or untreated Coronary Artery Disease (CAD), e.g. current angina/chest pain, a recent positive stress test, or significant stenosis that has not been revascularized?
  - Does the note mention the patient having an active systemic infection, e.g. sepsis, bacteremia, fungemia, or a plan to administer therapeutic (non-prophylactic) antibiotics?
  - Does the note mention the patient is currently an inpatient?
```

```
  - Does the note mention the expected surgical duration is prolonged, in that it lasts longer than 3 to 4 hours?
  - Does the note mention the surgery as having a high risk of needing a blood transfusion, e.g. cardiac surgery, major trauma surgery, or transplant surgery?
  - Does the note mention the patient having bleeding diathesis or coagulopathy, e.g. Hemophilia, von Willebrand disease, liver coagulopathy (INR > 1.5), or active use of therapeutic anticoagulants (e.g., Warfarin, Apixaban)?
  - Does the note mention the patient having low hemoglobin, e.g. anemia or hemoglobin < 10 g/dL?
  - Does the note mention the patient having severe baseline respiratory compromise, e.g. the use of home oxygen, home CPAP/BiPAP for reasons other than sleep apnea, or a history of tracheostomy?
  - Does the note mention the patient using of potentially nephrotoxic home medications, e.g. high-dose NSAIDs, ACE inhibitors, ARBs, chronic loop diuretics, or antibiotics like vancomycin or gentamicin?
* Other categories of concepts clinical experts have suggested considering include:
  - Patient characteristics: fluid accumulation, abnormal/ normal pulmonary findings, heart failure symptoms, acid reflux, bleeding disorders, abnormal laboratory values, creatinine levels, multiple medications, active infection requiring antibiotics, functional capacity (e.g., METS score, good exercise tolerance)
  - Pre-surgical status: location prior to surgery (ICU, hospital ward, or home), if the patient is intubated or needs supplemental oxygen
  - Surgical characteristics: anticipated bleeding risk, expected surgical duration, need for blood transfusion during the procedure, certain types of surgery that may be lower or higher risk for developing AKI, emergency surgery
  - Medication characteristics: multiple medications, nephrotoxic medications, antibiotics, insulin therapy

Propose at least ten candidates. Answer with a list of JSONs, one per line:
Finally, summarize all the generated candidates for the {num_concepts}-th meta-concept in a JSON. Merge any candidate meta-concepts that are essentially the same (where you would expect over 95% agreement) or essentially opposites (you would expect over 95% disagreement). In the JSON, include a list of comma-separated list of phrases that mean the same or opposite of each candidate meta-concept. The final JSON should have the following format:

Answer in the following JSON format:
{
  "reasoning": "Step-by-step explanation of how you systematically worked through the residual model concepts (from most to least predictive) and combined them with the feedback suggestions.",
  "concepts": [
    {
      "concept": "<QUESTION 1>",
      "is_risk_factor": <bool: True if a yes answer to this concept question is a risk factor, False if a yes answer to this concept question is a protective factor>,
      "words": ["<SYNONYM/ANTONYM 1>", "<SYNONYM/ANTONYM 2>", "<SYNONYM/ANTONYM 3>"]
    },
    {
      "concept": "<QUESTION 2>",
      "is_risk_factor": <bool: True if a yes answer to this concept question is a risk factor, False if a yes answer to this concept question is a protective factor>,
      "words": ["<SYNONYM/ANTONYM 1>", "<SYNONYM/ANTONYM 2>", "<SYNONYM/ANTONYM 3>"]
    },
    ...
    {
      "concept": "<QUESTION 10>",
      "is_risk_factor": <bool: True if a yes answer to this concept question is a risk factor, False if a yes answer to this concept question is a protective factor>,
      "words": ["<SYNONYM/ANTONYM 1>", "<SYNONYM/ANTONYM 2>", "<SYNONYM/ANTONYM 3>"]
    }
  ]
}


Example answer:
{
  "reasoning": "Step-by-step reasoning through the residual model concepts and feedback",
  "concepts": [
    {
      "concept": "Does the note mention the patient having signs of hepatic fluid accumulation, e.g., ascites, significant abdominal distension, or need for paracentesis?",
      "is_risk_factor": True,
      "words": [\ascites", \abdominal distension"]
    },
    {
      "concept": "Does the note mention the patient having normal pulmonary function, e.g., documentation of lungs being clear to auscultation?",
      "is_risk_factor": False,
      "words": [\clear lungs", \wheezing, \lungs clear"]
    },
    {
      "concept": "Does the note mention the patient having poor functional capacity, e.g., having less than 4 METs and inability to climb stairs?",
```

```
      "is_risk_factor": True,
      "words": ["unable to climb stairs", "poor exercise tolerance", "< 4 METs"]
    },
    {
      "concept": "Does the note mention the surgery having a high risk of major blood loss, e.g., open abdominal
      ↪  aortic aneurysm repair, liver transplantation, Whipple procedure, or anticipated blood loss (EBL) > 500mL?",
      "is_risk_factor": True,
      "words": ["open abdominal aortic aneurysm repair", "anticipated blood loss (EBL) > 500mL"]
    },
    {
      "concept": "Does the note mention the patient using of potentially nephrotoxic home medications, e.g., high-dose
      ↪  NSAIDs, ACE inhibitors, ARBs, chronic loop diuretics, or antibiotics like vancomycin or gentamicin?",
      "is_risk_factor": True,
      "words": ["high-dose NSAIDs", "ACE inhibitors", "ARBs", "chronic loop diuretics", "vancomycin", "gentamicin"]
    }
    ...
    ]
}
```

### F.3.4 ConceptAnnotationPrompt

```
You will be given clinical notes and a series of yes/no questions. For each question, answer with 1 (yes) if the
↪  information is mentioned in the note, or 0 (no) if it is not. Provide your answers in JSON format with each
↪  question mapped to its corresponding answer (0 or 1) Questions:
{prompt_questions}

clinical notes:
{sentence}

Example answer: {
    "0": 0,
    "1": 1,
    "2": 0
}
Answer all the questions and do not answer with anything else besides valid JSON. Do not add comments to the JSON.
```
